# Supplementary material for: Nonoperative treatment versus volar locking plating for distal radius fracture in patients aged 65 years or older (DRIFT trial): A randomized controlled trial
Source: PLoS Med. 2025 Sep 5;22(9):e1004728. doi: 10.1371/journal.pmed.1004728 (PMC12425212; doi:10.1371/journal.pmed.1004728)
Supplement: S1 Text — (DOCX) [file pmed.1004728.s003.docx]

**Värttinäluun alaosan murtuma eli rannemurtuma ja kipsihoito**

Tämä potilasohje on koottu tukemaan värttinäluun alaosan murtumasta eli rannemurtumasta kuntoutumista. Ohje on tarkoitettu niille, joiden murtuma on hoidettu kipsaamalla eli konservatiivisesti. Ohje sisältää yleistä tietoa murtumasta sekä ranteen kuntouttamisen kotiharjoitteluohjeet.

Värttinäluun distaalinen eli luun alaosan murtuma on yleisin yläraajamurtuma. Tyypillisesti murtuma syntyy kaaduttaessa ojennetun käden varaan. Erityisesti talven liukkaat sääolosuhteet ovat suurena riskinä kaatumisille ja värttinäluun alaosan murtumille.


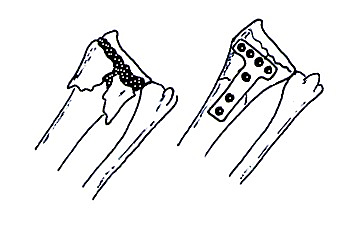


**Omatoimisen harjoittelun tärkeys**

Ensisijaisena tavoitteena kipsauksen jälkeen on palauttaa ranteen normaali liikkuvuus. Edellytyksenä tavoitteen saavuttamiseksi on ohjeen mukainen **säännöllinen** harjoittelu, joka on ranteen toimintakyvyn kannalta hyvin tärkeää. Harjoitteiden aloittamista ei tule missään nimessä viivästyttää eikä kipsihoidon jälkeistä ranteen liikuttelua tule turhaan pelätä. Tämän ohjevihkon harjoitteet on suunniteltu paranemisaikataulua kunnioittaen ja ovat siksi turvallisia toteuttaa.

Ahkera ja pitkäjänteinen omatoiminen harjoittelu näkyy parhaimmillaan ranteen palautuneena toimintakykynä. Harjoittelematta jättäminen tai harjoitusten viivästyttäminen voi pahimmillaan johtaa jäykkään ja toimintakyvyltään heikkoon ranteeseen.

- Aloita rohkeasti ohjeen mukainen harjoittelu.
- Ahkera harjoittelu mahdollistaa hyvän lopputuloksen.
- Vammautunutta kättä kannattaa käyttää alusta saakka kevyissä arkiaskareissa, kuten ruokaillessa ja hampaiden pesussa.
- Harjoittele kärsivällisesti. Ranteen toimintakyvyn palautuminen ei tapahdu hetkessä.
- Noudata harjoitusaikataulua, mutta kunnioita epämiellyttäviä tuntemuksiasi!

**Kipsihoidon aikaiset harjoitteet**

Rannetta on suojaamassa kipsi viiden viikon ajan. Murtuman jälkeen on normaalia, että ranne on kipeä ja siinä esiintyy turvotusta. Tavoitteet heti kipsauksen jälkeen keskittyvätkin turvotuksen lievitykseen ja kivun hoitoon.

**1. Kohoasento**

Niin kauan kuin turvotusta ilmenee, kannattaa kättä pitää paljon kohoasennossa sydämen tason yläpuolella. Kohoasento helpottaa sekä kipua että turvotusta. Kantosidettä ei tule käyttää.

Tue levätessäsi käsi kohoasentoon tyynyillä ja liikkuessasi pidä kättä rintakehän päällä. Kun turvotus ei enää ole häiritsevää, on tärkeää, että annat käden olla vapaana ja vältät sen turhaa kannattelua.


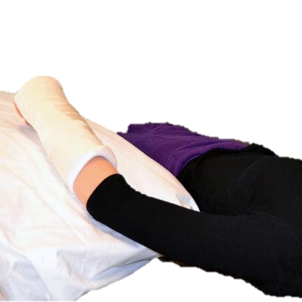

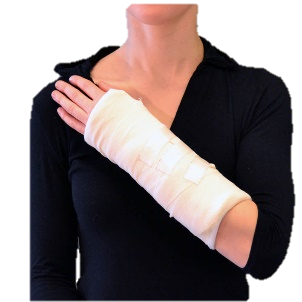

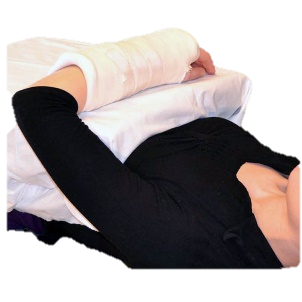


**2. Kylmähoito**

Kylmähoito rauhoittaa kudoksia ja on tehokas kivun hoidossa. Toteuta säännöllistä kylmähoitoa kotona kylmäpakkauksin. Pidä kylmäpakkausta ranteen kämmenen puolella lyhyitä aikoja useasti päivässä. Varo palelluttamasta ihoa. Kylmäpakkausta ei tule koskaan pitää paljasta ihoa vasten.

**3. Sormien harjoitukset**

Aloita sormien liikeharjoitukset heti kipsauksen jälkeen. Tee seuraavia harjoituksia hereillä ollessasi noin **tunnin välein**. **Sormien liikuttelua et voi tehdä liikaa**. Liikkeet vähentävät käden turvotusta ja kipua.

1. Vie sormet täysin nyrkkiin niin, että peukalon jää päällimmäiseksi. Ojenna sormet sen jälkeen aivan suoriksi ja erilleen toisistaan niin leveälle kuin mahdollista. Jos et pysty tekemään liikettä alkuun täydellä aktiivisella liikeradalla, voit avustaa kevyesti toisella kädellä niin kauan, että täysi aktiivinen liike onnistuu. Toista liike 10 kertaa.


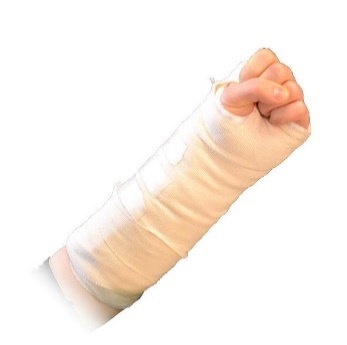


***a***


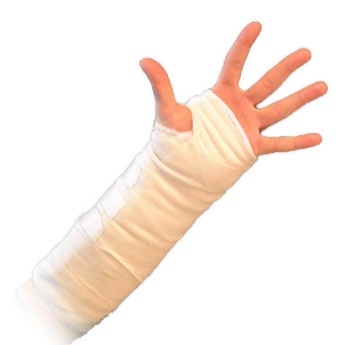


1. Kosketa peukalolla vuorotellen jokaista sormenpäätä. Jokaisen kosketuksen jälkeen ojenna sormet suoraksi, aivan kuten nyrkistyksen jälkeen. Toista liikettä niin, että kosketat vuoronperää jokaista sormea 10 kertaa.

1. Pyöritä peukalolla suurta ympyrää. Tee liikettä 10 kierrosta kumpaankin suuntaan.


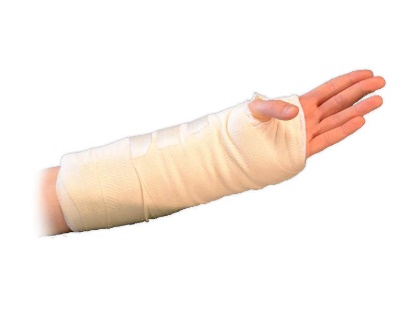

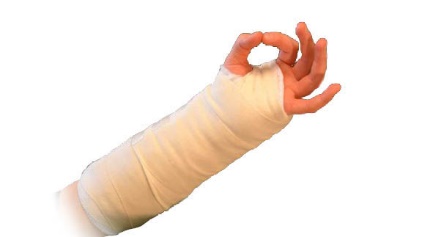


***b***

***c***

**4. Koko yläraajan liikeharjoitukset**

Ranteen ollessa kipsissä on vaarana, että koko yläraaja jää käyttämättömäksi. Olkanivelesi ja kyynärnivelesi tarvitsevat kipsistä huolimatta liikettä. Tee siis seuraavia harjoituksia useasti päivässä.

1. Pidä käsi vartalon vieressä. Ojenna kyynärnivel täysin suoraksi ja ojenna samalla myös sormet. Pidä käsivartta hetki aivan suorana. Koukista sitten kyynärnivel niin koukkuun kuin saat ja koukista samalla myös sormet. Kipsin ei tulisi estää koukistusta. Tee liikettä 10 kertaa ja toista useita kertoja päivässä.
2. Nosta käsi kohti kattoa. Muista hyvä ryhti liikettä tehdessäsi. Tee liikettä 10 kertaa ja toista useita kertoja päivässä.

Voit tehdä olkanivelen liikeharjoitetta myös makuuasennossa nostamalla kättä pääsi yläpuolelle tyynylle.


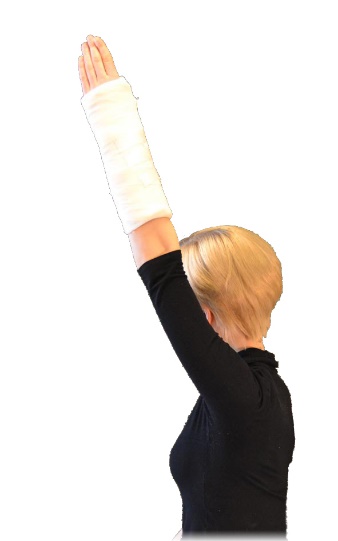


***a***


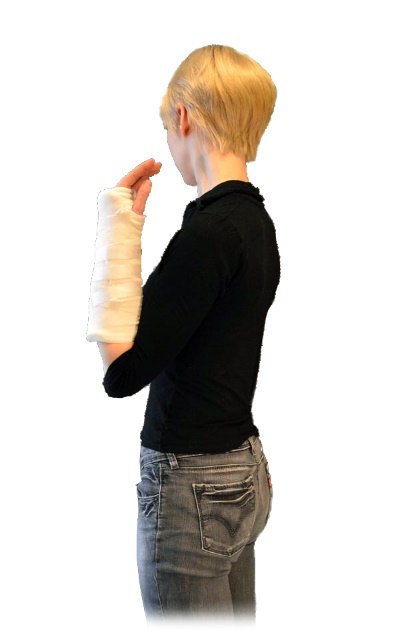

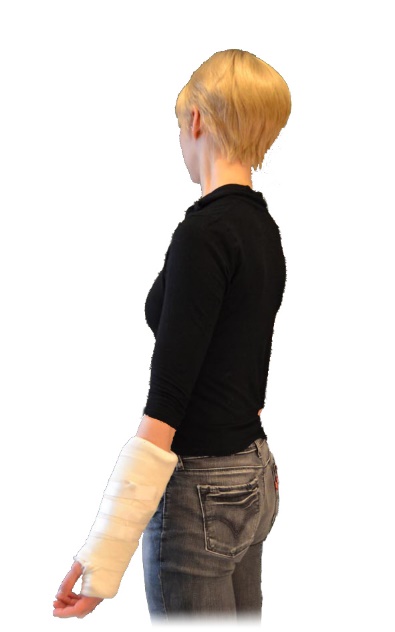


***b***

**Kipsin poiston jälkeen**

Kipsi poistetaan noin viiden viikon kuluttua, jolloin on myös ranteen röntgenkuvaus ja lääkärikontrolli. Kontrollissa varmistetaan, että murtuma on luutunut odotetusti. Jos kaikki on kunnossa, voit aloittaa käden normaalin käytön. Käden ja ranteen käyttö arjen normaaleissa toiminnoissa on tärkeää, sillä se edesauttaa toimintakyvyn palautumista.

Nyt on myös aika aloittaa ranteen ahkera liikeharjoittelu. Säännöllisen harjoittelun avulla voidaan palauttaa ranteen toimintakyky mahdollisimman hyväksi. Ranne voi olla alkuun hyvin jäykkä ja ensisijainen tavoite välittömästi kipsin poiston jälkeen onkin liikkuvuuden lisääminen. Tee harjoituksia useita kertoja päivän aikana.

**1. Ranteen ja kyynärvarren liikeharjoitukset**

Tukemalla kyynärvartesi pöytää vasten varmistut siitä, että kyynärvarsi pysyy paikallaan ja saat tehtyä liikkeet mahdollisimman laajasti ja puhtaasti.

1. Tee harjoitus kämmensyrjä pöytää vasten. Sormet saavat olla kevyesti koukussa. Ojenna ja koukista rannettasi rauhallisesti. Tee mahdollisimman suuri liike. Tee liikettä 10 kertaa molempiin suuntiin.

***a***


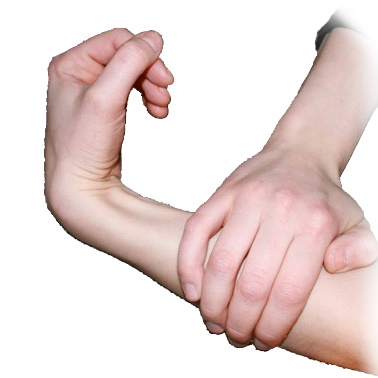

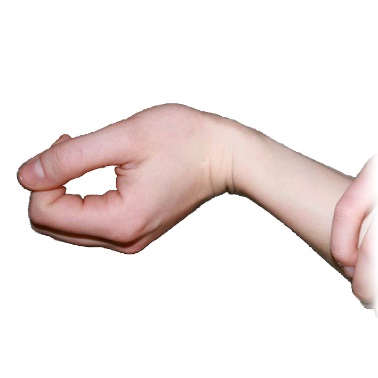


1. Käännä kämmen pöytää vasten. Sormet saavat olla rentoina. Tee sivusuuntaista hidasta liikettä (ikään kuin pyyhkisit pöydän pintaa) niin suurena kuin mahdollista. Tee liikettä 10 kertaa molempiin suuntiin.

***b***


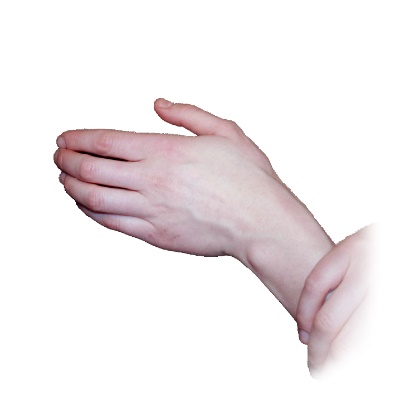

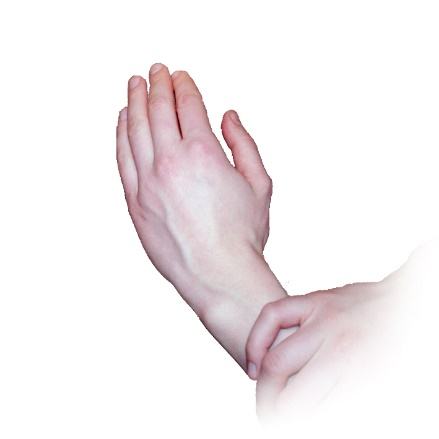


1. Istu ryhdikkäässä asennossa olkavarsi vartalosi vieressä ja kyynärnivel noin 90 asteen koukussa. Käännä vuorotellen kämmen ja kämmenselkä kohti kattoa. Tee käden kääntö 10 kertaa molempiin suuntiin.


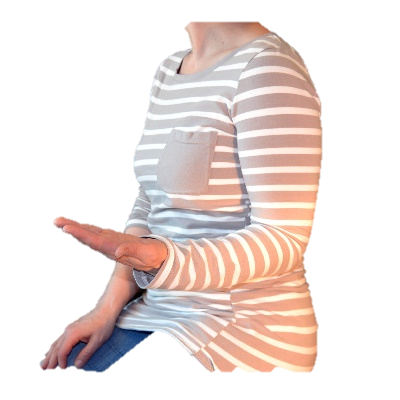

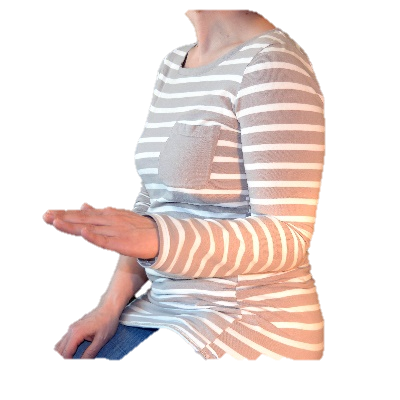


***c***

***c***

**2. Pehmeän lankarullan/ sideharsorullan puristus**

Pidä pehmeää käteen sopivaa lankarullaa tai esimerkiksi sideharsorullaa kevyesti kädessäsi. Purista esinettä 5 sekunnin ajan ja hellitä puristus. Toista harjoitus 10 kertaa.


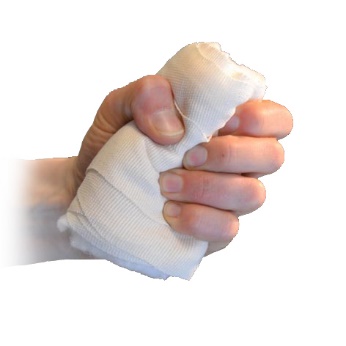


**Kun edelliset liikeharjoitukset sujuvat hyvin siirry harjoittelemaan painovoima vastuksena.** Liikkuvuuden lisäämisen lisäksi liikkeet aktivoivat ranteen lihastoimintaa.

**3. Liikeharjoitus pöydän reunalta**

1. Pidä ranteesi pöydän reunalla kämmenselkä kattoa kohti niin, että kätesi voi liikkua vapaasti. Sormet saavat olla kevyesti koukussa. Liikuta kättä hitaasti itseäsi kohden kämmenselkä edellä. Palauta liike hitaasti alas. Toista harjoitus 10 kertaa.


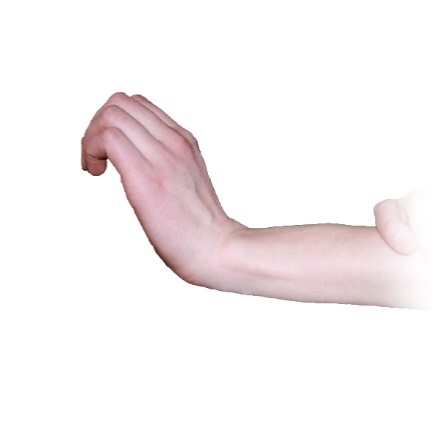


***a***

1. Käännä kätesi niin, että peukalosi on kohti kattoa. Sormet voivat olla edelleen kevyesti koukussa. Käännä kättäsi hitaasti pyrkien osoittamaan peukalolla itseäsi. Palauta käsi hitaasti takaisin alkuasentoon. Toista harjoitus 10 kertaa.


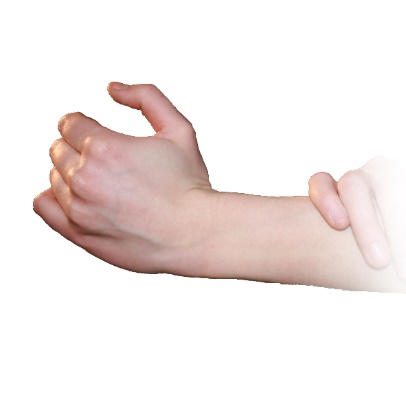


***b***

1. Käännä kämmen kohti kattoa. Sormet saavat olla kevyesti koukussa. Käännä kämmentä itseäsi kohti. Palauta käsi hitaasti takaisin alas. Toista harjoitus 10 kertaa.


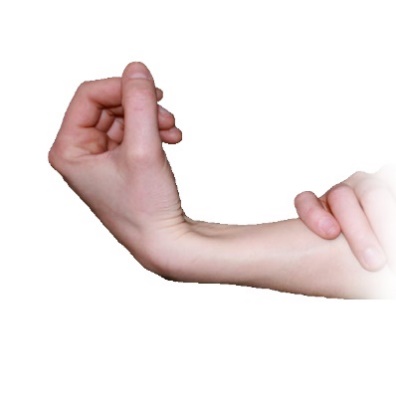


***c***

**7 viikkoa vammasta**

Ranteen toimintakyvyn palautuminen on aina yksilöllistä. Ranne on pääosin palautunut kuormituskyvyn ja liikeratojen osalta noin kolmen kuukauden kuluttua vammasta. Täydellinen palautuminen voi kestää vuoden tai pidempään. Palautumiseen kuluva aika riippuu vamman vakavuudesta ja harjoitteluaktiivisuudesta. Aktiivinen ranteen liikeharjoittelu nopeuttaa vammasta kuntoutumista.

Jatka yhä aiempia harjoituksia, sillä säännöllinen liikkuvuusharjoittelu on edelleen tärkeää ranteen toimintakyvyn palauttamiseksi. Nyt harjoituksiin tulee mukaan ranteen venytys, joka edesauttaa liikkuvuuden lisääntymistä. Lisäksi edellisiltä viikoilta tuttua puristusliikettä muutetaan hieman.

**1. Pallon puristus**

Purista esimerkiksi tennispalloa 5 sekunnin ajan. Pallo saa olla nyt kovempi kuin edellisessä puristusharjoituksessa. Harjoituksesta tulee näin vahvistavampi. Toista puristus 10 kertaa.


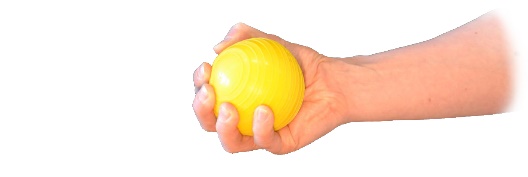


**2. Ranteen venytys**

1. Ojenna vammautunut ranne terveen käden avulla niin, että tunnet venytyksen ranteen kämmenen puolella. Venytä kevyesti 30 sekunnin ajan. Toista venytys.
2. Koukista vammautunut ranne terveellä kädellä. Venytys tuntuu nyt ranteen kämmenselän puolella. Venytä kevyesti 30 sekuntia. Toista venytys.


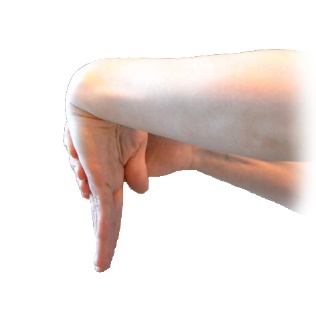

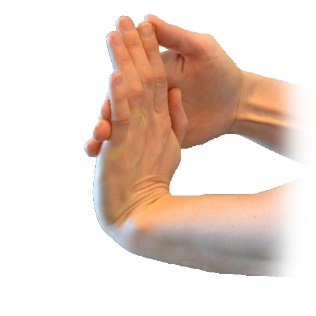


***a***

***b***

Jos ranteen paraneminen on edennyt odotetusti, voit ottaa harjoitteluun mukaan myös seuraavien sivujen kevyet lihasvoimaharjoitukset. Harjoitusten avulla käsi ja ranne saavat vähitellen tarvitsemaansa vahvistusta.

**3. Vastustettu lihasvoimaharjoitus**

Omalla kädellä vastustetut lihasvoimaharjoitukset ovat turvallisia tehdä. Seuraavissa harjoituksissa käsiä painetaan toisiaan vasten.

1. Paina terveellä kädellä vammautuneen käden kämmentä vasten. Vastusta liikettä vammautuneella kädellä niin, ettei käsi liiku. Paina käsiä toisiaan vasten 5 sekunnin ajan. Toista harjoitus 5 kertaa.


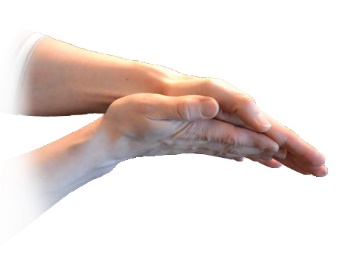


***a***

1. Paina terveellä kädellä vammautuneen käden kämmenselkää vasten. Vastusta liikettä vammautuneella kädellä niin, ettei käsi liiku. Paina käsiä toisiaan vasten 5 sekunnin ajan. Toista harjoitus 5 kertaa.

***b***


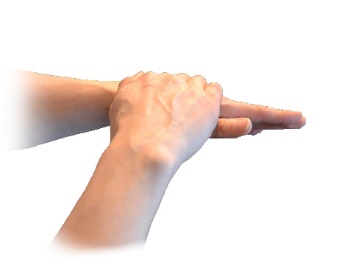


**9 viikkoa vammasta**

**1. Lihasvoimaharjoitus kevyellä painolla**

Seuraavat liikkeet voit tehdä kevyellä käsipainolla (0,5 kg) tai käyttäen painona esimerkiksi 1/2 litran juomapulloa. Liikkeet vahvistavat rannetta.

1. Pidä ranteesi pöydän reunalla kämmenselkä kattoa kohti niin, että kätesi voi liikkua vapaasti. Pidä paino kädessäsi. Nosta painoa kääntäen kämmenselkää itseäsi kohti. Palauta käsi hitaasti alas. Toista liike 10 kertaa.
2. Käännä paino kuvan mukaisesti pystyasentoon. Nosta painoa kääntäen sen yläosaa itseäsi kohti. Muista taas käden hidas palautus takaisin alkuasentoon. Toista 10 kertaa.
3. Käännä paino niin, että kämmenesi on kattoa kohti. Nosta painoa kääntäen kämmentä itseäsi kohti. Palauta käsi hitaasti takaisin alas. Toista 10 kertaa.


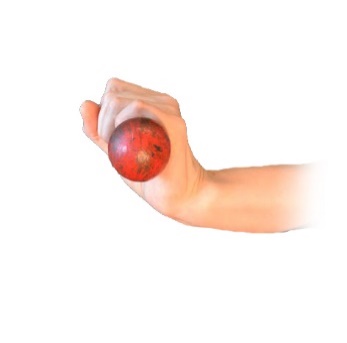


***a***


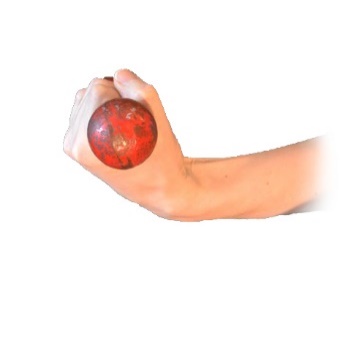


***c***


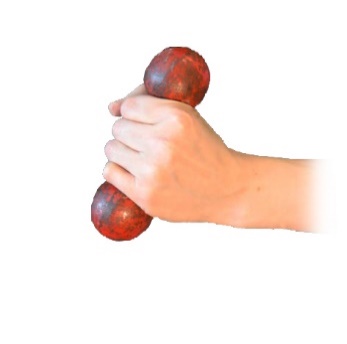


***b***

**Ranteen vähitellen palautuva toimintakyky on palkkio säännöllisesti toteutetusta omatoimisesta harjoittelusta!**

**Ohjevihkon toteutus ja valokuvat:**

Ohjevihko pohjautuu Niina Maijalan ja Annariina Ruuskasen opinnäytetyöhön Kuntoutuminen volaarisella levyllä kiinnitetystä värttinäluun murtumasta. Kotiharjoitteluohjeita rannemurtumapotilaalle.

Potilasohje on toteutettu yhteistyössä TAYS:n fysiatrian yksikön henkilökunnan kanssa.

Fysioterapian koulutusohjelma

Syksy 2013

Marianne Suurkari, ft, TtM

Antti Launonen, LT, ortopedian ja traumatologian erikoislääkäri, käsikirurgi

**Värttinäluun alaosan murtuma eli rannemurtuma ja leikkaushoito**

Tämä potilasohje on koottu tukemaan värttinäluun murtumasta eli rannemurtumasta kuntoutumista. Ohje on tarkoitettu niille, joiden murtuma on hoidettu leikkauksella. Ohje sisältää yleistä tietoa murtumasta sekä ranteen kuntouttamisen kotiharjoitteluohjeet.

Värttinäluun distaalinen eli luun alaosan murtuma on yleisin yläraajamurtuma. Tyypillisesti murtuma syntyy kaaduttaessa ojennetun käden varaan. Erityisesti talven liukkaat sääolosuhteet ovat suurena riskinä kaatumisille ja värttinäluun alaosan murtumille.

**Värttinäluun alaosan murtuman leikkaushoito**

Käytetyin leikkausmenetelmä värttinäluun alaosan murtumien hoidossa on niin sanotun volaarisen eli kämmenen puoleisen lukkolevyn käyttö. Murtuma asetetaan leikkauksessa paikalleen ja kiinnitetään tukevasti lukkolevyllä. Etuna lukkolevyn käytössä on se, että ranteen kuntouttavat harjoitteet voidaan aloittaa heti kahden viikon kipsihoidon jälkeen. Ilman leikkausta kipsihoito on pidempi.


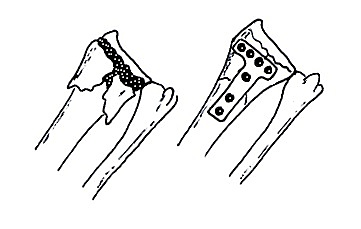


**Omatoimisen harjoittelun tärkeys**

Ensisijaisena tavoitteena leikkauksen jälkeen on palauttaa ranteen normaali liikkuvuus. Edellytyksenä tavoitteen saavuttamiseksi on ohjeen mukainen **säännöllinen** harjoittelu, joka on ranteen toimintakyvyn kannalta hyvin tärkeää. Harjoitteiden aloittamista ei tule missään nimessä viivästyttää eikä kipsihoidon jälkeistä ranteen liikuttelua tule turhaan pelätä. Tämän ohjevihkon harjoitteet on suunniteltu paranemisaikataulua kunnioittaen ja ovat siksi turvallisia toteuttaa.

Ahkera ja pitkäjänteinen omatoiminen harjoittelu näkyy parhaimmillaan ranteen palautuneena toimintakykynä. Harjoittelematta jättäminen tai harjoitusten viivästyttäminen voi pahimmillaan johtaa jäykkään ja toimintakyvyltään heikkoon ranteeseen.

- Aloita rohkeasti ohjeen mukainen harjoittelu.
- Ahkera harjoittelu mahdollistaa hyvän lopputuloksen.
- Leikattua kättä kannattaa käyttää alusta saakka kevyissä arkiaskareissa, kuten ruokaillessa ja hampaiden pesussa.
- Harjoittele kärsivällisesti. Ranteen toimintakyvyn palautuminen ei tapahdu hetkessä.
- Noudata harjoitusaikataulua, mutta kunnioita epämiellyttäviä tuntemuksiasi!

**Kipsihoidon aikaiset harjoitteet**

Leikkauksen jälkeen rannetta on suojaamassa kipsi kahden viikon ajan. Toimenpiteen jälkeen on normaalia, että ranne on kipeä ja siinä esiintyy turvotusta. Tavoitteet heti leikkauksen jälkeen keskittyvätkin turvotuksen lievitykseen ja kivun hoitoon.

**1. Kohoasento**

Niin kauan kuin turvotusta ilmenee, kannattaa kättä pitää paljon kohoasennossa sydämen tason yläpuolella. Kohoasento helpottaa sekä kipua että turvotusta. Kantosidettä ei leikkauksen jälkeisessä hoidossa käytetä.

Tue levätessäsi käsi kohoasentoon tyynyillä ja liikkuessasi pidä kättä rintakehän päällä. Kun turvotus ei enää ole häiritsevää, on tärkeää, että annat käden olla vapaana ja vältät sen turhaa kannattelua.


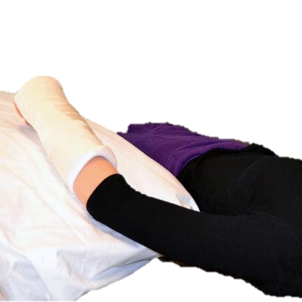

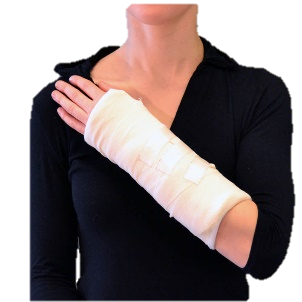

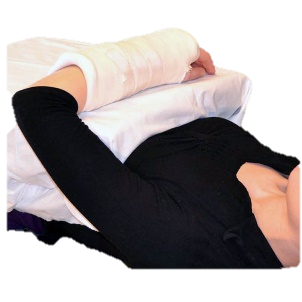


**2. Kylmähoito**

Kylmähoito rauhoittaa kudoksia leikkauksen jälkeen ja on tehokas kivun hoidossa. Toteuta säännöllistä kylmähoitoa kotona kylmäpakkauksin. Aloita kylmähoito välittömästi leikkauksen jälkeen. Pidä kylmäpakkausta ranteen kämmenen puolella lyhyitä aikoja useasti päivässä. Varo palelluttamasta ihoa. Kylmäpakkausta ei tule koskaan pitää paljasta ihoa vasten.

**3. Sormien harjoitukset**

Aloita sormien liikeharjoitukset heti leikkauksen jälkeen. Tee seuraavia harjoituksia hereillä ollessasi noin **tunnin välein. Sormien liikuttelua et voi tehdä liikaa**. Liikkeet vähentävät käden turvotusta ja kipua.

1. Vie sormet täysin nyrkkiin niin, että peukalo jää päällimmäiseksi. Ojenna sormet sen jälkeen aivan suoriksi ja erilleen toisistaan niin leveälle kuin mahdollista. Jos et pysty tekemään liikettä alkuun täydellä aktiivisella liikeradalla, voit avustaa kevyesti toisella kädellä niin kauan, että täysi aktiivinen liike onnistuu. Toista liike 10 kertaa


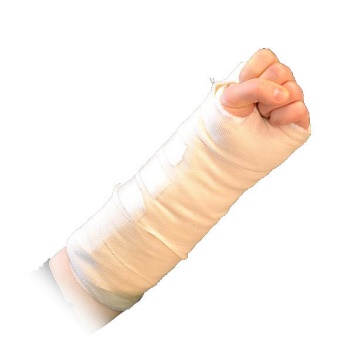


***a***


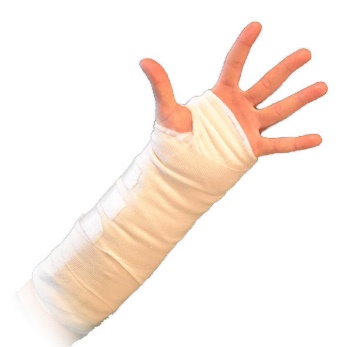


1. Kosketa peukalolla vuorotellen jokaista sormenpäätä. Jokaisen kosketuksen jälkeen ojenna sormet suoraksi, aivan kuten nyrkistyksen jälkeen. Toista liikettä niin, että kosketat vuoronperää jokaista sormea 10 kertaa.

1. Pyöritä peukalolla suurta ympyrää. Tee liikettä 10 kierrosta kumpaankin suuntaan.


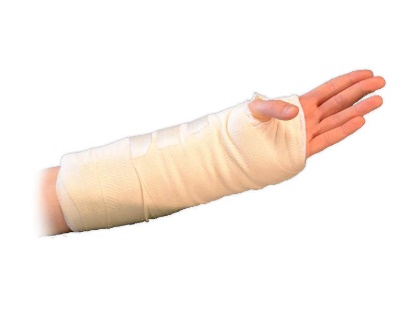

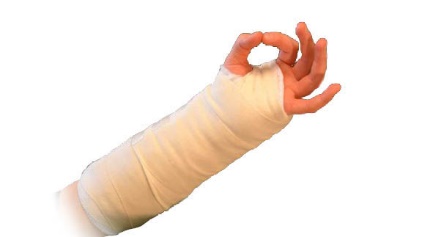


***b***

***c***

**4. Koko yläraajan liikeharjoitukset**

Ranteen ollessa kipsissä on vaarana, että koko yläraaja jää käyttämättömäksi. Olkanivelesi ja kyynärnivelesi tarvitsevat kipsistä huolimatta liikettä. Tee siis seuraavia harjoitteita useasti päivässä.

1. Pidä käsi vartalon vieressä. Ojenna kyynärnivel täysin suoraksi ja ojenna samalla myös sormet. Pidä käsivartta hetki aivan suorana. Koukista sitten kyynärnivel niin koukkuun kuin saat ja koukista samalla myös sormet. Kipsin ei tulisi estää koukistusta. Tee liikettä 10 kertaa ja toista useita kertoja päivässä.
2. Nosta käsi kohti kattoa. Muista hyvä ryhti liikettä tehdessäsi. Tee liikettä 10 kertaa ja toista useita kertoja päivässä.

Voit tehdä olkanivelen liikeharjoitetta myös makuuasennossa nostamalla kättä pääsi yläpuolelle tyynylle.


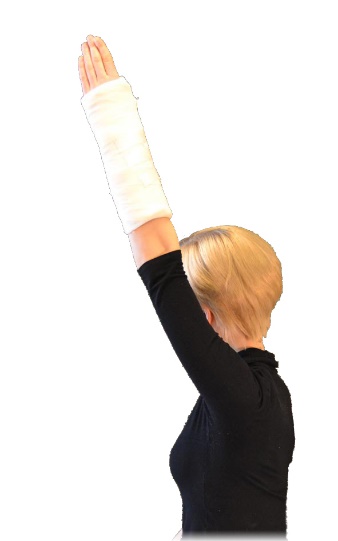


***a***


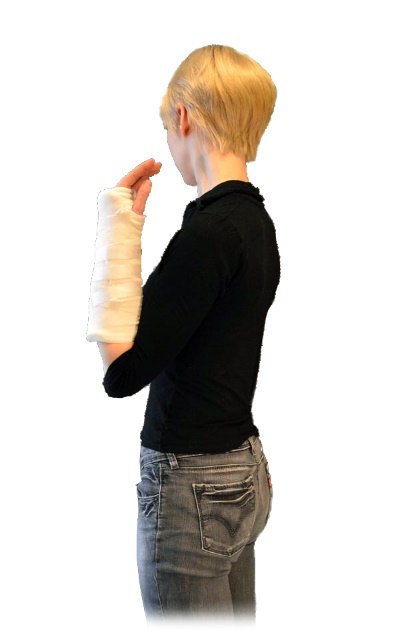

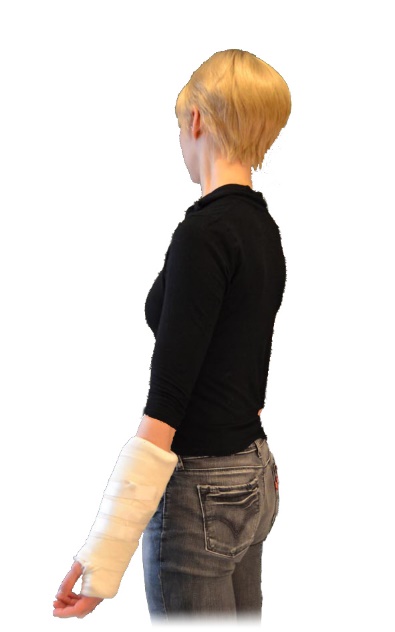


***b***

**Kipsin poiston jälkeen**

Kipsi poistetaan kahden viikon kuluttua leikkauksesta ja tällöin on aika aloittaa ranteen ahkera liikeharjoittelu. Säännöllisen harjoittelun avulla voidaan palauttaa ranteen toimintakyky mahdollisimman hyväksi. Ranne voi olla alkuun hyvin jäykkä ja ensisijainen tavoite välittömästi kipsin poiston jälkeen onkin liikkuvuuden lisääminen. Tee harjoitteita useita kertoja päivän aikana.

**1. Ranteen ja kyynärvarren liikeharjoitteet**

Liikkuvuuden lisäämisen lisäksi liikkeet aktivoivat ranteen lihastoimintaa. Jos liikkeet a ja b tuntuvat alkuun liian raskailta, voit tehdä liikkeen pöydän tasossa painovoima eliminoituna kuten kuvassa c, mutta peukalo ylöspäin.

1. Pidä ranteesi pöydän reunalla kämmenselkä kattoa kohti niin, että kätesi voi liikkua vapaasti. Sormet saavat olla kevyesti koukussa. Liikuta kättä hitaasti itseäsi kohden kämmenselkä edellä. Palauta liike hitaasti alas. Toista harjoitus 10 kertaa.


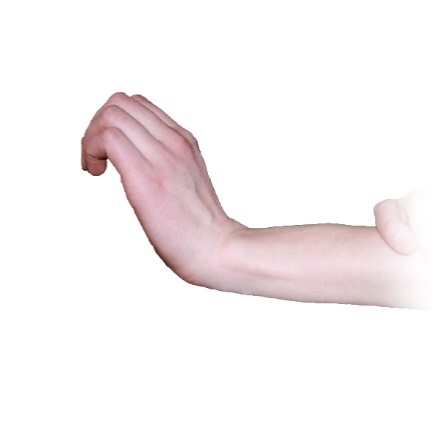


***a***

1. Käännä kämmen kohti kattoa. Sormet saavat olla kevyesti koukussa. Käännä kämmentä itseäsi kohti. Palauta käsi hitaasti takaisin alas. Toista harjoitus 10 kertaa.


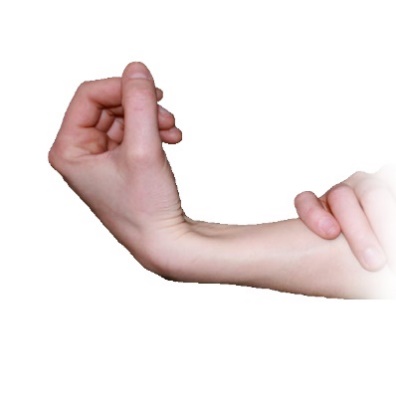


***b***

1. Käännä kämmen pöytää vasten ja pidä toisen käden tuki kyynärvarressasi. Sormet saavat olla rentoina. Tee sivusuuntaista hidasta liikettä (ikään kuin pyyhkisit pöydän pintaa) niin suurena kuin mahdollista. Edistymisen myötä voit tehdä harjoituksen pöydän reunan yli peukalo ylöspäin. Tee liikettä 10 kertaa molempiin suuntiin.

***c*)**


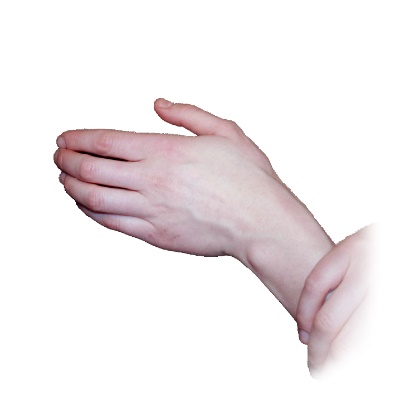

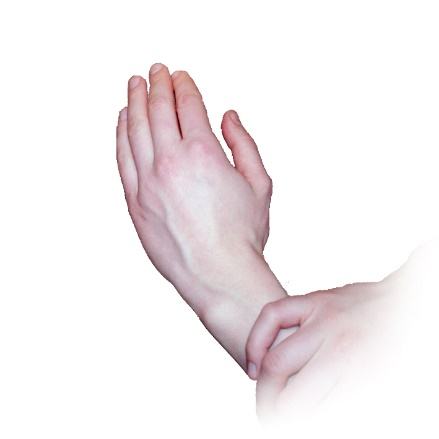


1. Istu ryhdikkäässä asennossa olkavarsi vartalosi vieressä ja kyynärnivel noin 90 asteen koukussa. Käännä vuorotellen kämmen ja kämmenselkä kohti kattoa. Tee käden kääntö 10 kertaa molempiin suuntiin.

***d***


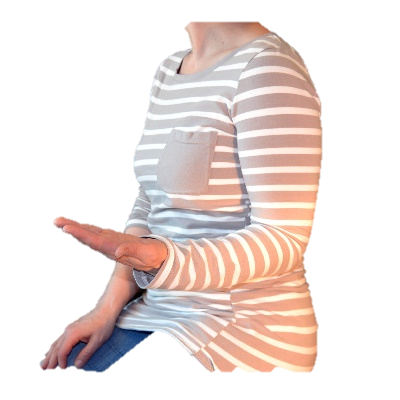

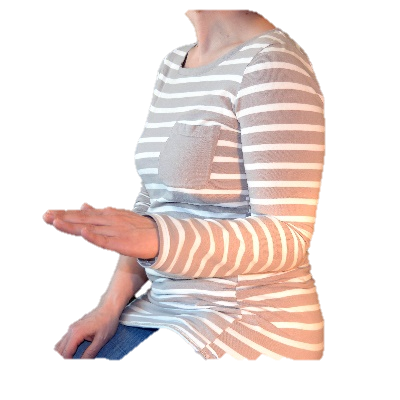


**2. Pehmeän lankarullan/ sideharsorullan puristus**


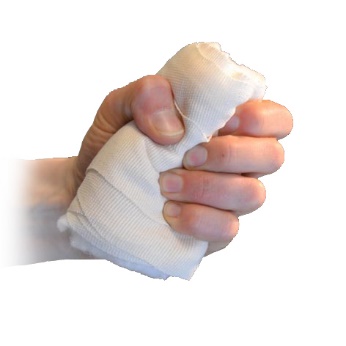
Pidä pehmeää käteen sopivaa lankarullaa tai esimerkiksi sideharsorullaa kevyesti kädessäsi. Purista esinettä 5 sekunnin ajan ja hellitä puristus. Toista harjoitus 10 kertaa.

**5 viikkoa leikkauksesta**

Viiden viikon kuluttua leikkauksesta on leikatun ranteen röntgenkuvaus ja lääkärikontrolli. Kontrollissa varmistetaan, että murtuma on luutunut odotetusti. Jos kaikki on kunnossa, voit aloittaa leikatun käden normaalin käytön. Käden ja ranteen käyttö arjen normaaleissa toiminnoissa on tärkeää, sillä se edesauttaa toimintakyvyn palautumista.

Ranteen toimintakyvyn palautuminen on aina yksilöllistä. Ranne on pääosin palautunut kuormituskyvyn ja liikeratojen osalta noin kolmen kuukauden kuluttua leikkauksesta. Täydellinen palautuminen voi kestää vuoden tai pidempään. Palautumiseen kuluva aika riippuu vamman vakavuudesta ja harjoitteluaktiivisuudesta. Varhain aloitettu ja aktiviinen ranteen liikeharjoittelu nopeuttaa vammasta kuntoutumista.

Jatka yhä aiempia harjoituksia, sillä säännöllinen liikkuvuusharjoittelu on edelleen tärkeää ranteen toimintakyvyn palauttamiseksi. Nyt harjoituksiin tulee mukaan ranteen venytys, joka edesauttaa liikkuvuuden lisääntymistä sekä vastustettu lihasvoimaharjoitus. Lisäksi edellisiltä viikoilta tuttua puristusliikettä muutetaan hieman.

**1. Pallon puristus**


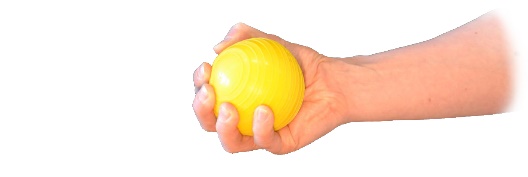
Purista esimerkiksi tennispalloa 5 sekunnin ajan. Pallo saa olla nyt kovempi kuin edellisessä puristusharjoituksessa. Harjoituksesta tulee näin vahvistavampi. Toista puristus 10 kertaa.

**2. Ranteen venytys**

1. Ojenna leikattu ranne terveen käden avulla niin, että tunnet venytyksen ranteen kämmenen puolella. Venytä kevyesti 30 sekunnin ajan. Toista venytys.
2. Koukista leikattu ranne terveellä kädellä. Venytys tuntuu nyt ranteen kämmenselän puolella. Venytä kevyesti 30 sekuntia. Toista venytys.


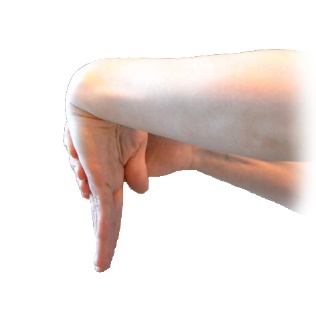

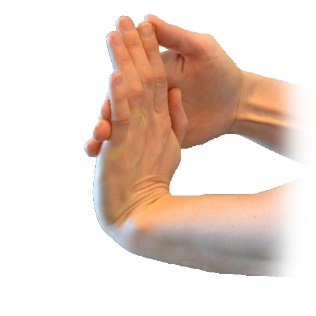


***a***

***b***

**b)**

**3. Vastustettu lihasvoimaharjoitus**

Omalla kädellä vastustetut lihasvoimaharjoitukset ovat turvallisia tehdä. Seuraavissa harjoituksissa käsiä painetaan toisiaan vasten.

1. Paina terveellä kädellä leikatun käden kämmentä vasten. Vastusta liikettä leikatulla kädellä niin, ettei käsi liiku. Paina käsiä toisiaan vasten 5 sekunnin ajan. Toista harjoitus 5 kertaa.
2. Paina terveellä kädellä leikatun käden kämmenselkää vasten. Vastusta liikettä leikatulla kädellä niin, ettei käsi liiku. Paina käsiä toisiaan vasten 5 sekunnin ajan. Toista harjoitus 5 kertaa.


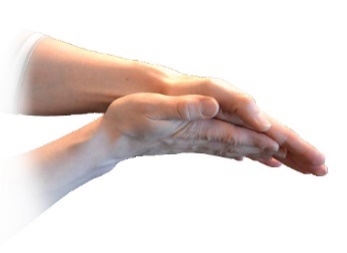


***a***

***b***


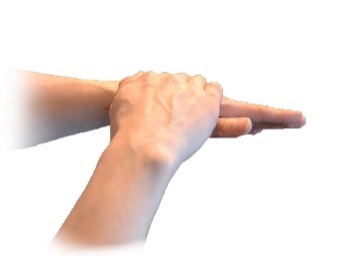


**6 viikkoa leikkauksesta**

Jatka edelleen kotiharjoitteiden ahkeraa ja säännöllistä tekemistä. Monipuolinen liikkuvuusharjoittelu on yhä tärkeää ranteen toimintakyvylle. Jos ranteen paraneminen on edennyt odotetusti, voit ottaa harjoitteluun mukaan kevyen painon.

**1. Lihasvoimaharjoitus kevyellä painolla**

Seuraavat liikkeet voit tehdä kevyellä käsipainolla (0,5 kg) tai käyttäen painona esimerkiksi 1/2 litran juomapulloa. Liikkeet vahvistavat rannetta.

1. Pidä ranteesi pöydän reunalla kämmenselkä kattoa kohti niin, että kätesi voi liikkua vapaasti. Pidä paino kädessäsi. Nosta painoa kääntäen kämmenselkää itseäsi kohti. Palauta käsi hitaasti alas. Toista liike 10 kertaa.
2. Käännä paino kuvan mukaisesti pystyasentoon. Nosta painoa kääntäen sen yläosaa itseäsi kohti. Muista taas käden hidas palautus takaisin alkuasentoon. Toista 10 kertaa.
3. Käännä paino niin, että kämmenesi on kattoa kohti. Nosta painoa kääntäen kämmentä itseäsi kohti. Palauta käsi hitaasti takaisin alas. Toista 10 kertaa.


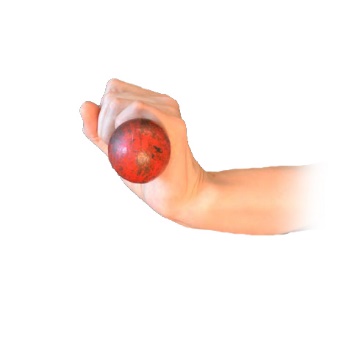


***a***


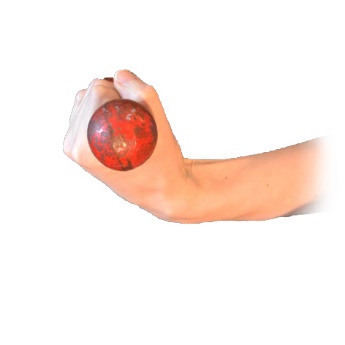


***c***


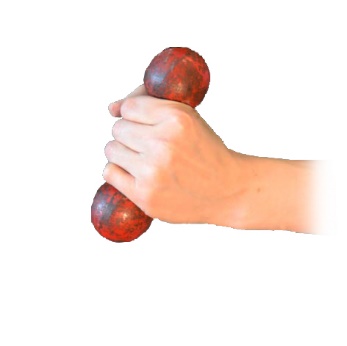


***b***

**Ranteen vähitellen palautuva toimintakyky on palkkio säännöllisesti toteutetusta omatoimisesta harjoittelusta!**

**Ohjevihkon alkuperäinen toteutus ja valokuvat:**

Ohjevihko pohjautuu Niina Maijalan ja Annariina Ruuskasen opinnäytetyöhön Kuntoutuminen volaarisella levyllä kiinnitetystä värttinäluun murtumasta. Kotiharjoitteluohjeita rannemurtumapotilaalle.

Potilasohje on toteutettu yhteistyössä TAYS:n fysiatrian yksikön henkilökunnan kanssa.

Fysioterapian koulutusohjelma

Syksy 2013

Marianne Suurkari, ft, TtM

Antti Launonen, LT, ortopedian ja traumatologian erikoislääkäri, käsikirurgi
