# Supplementary material for: Nonoperative treatment versus volar locking plating for distal radius fracture in patients aged 65 years or older (DRIFT trial): A randomized controlled trial
Source: PLoS Med. 2025 Sep 5;22(9):e1004728. doi: 10.1371/journal.pmed.1004728 (PMC12425212; doi:10.1371/journal.pmed.1004728)
Supplement: S2 Text — (PDF) [file pmed.1004728.s004.pdf]

Date: 13.5.24
Version: 1.1

Statistical analysis plan (SAP)

Nordic Innovative Trial to Evaluate OsteoPorotic Fractures (NITEP-group)
DRIFT trial: Non-operative treatment versus surgery with volar locking plate in the treatment of distal radius fracture in patients aged 65 and over – a prospective, randomized controlled trial

Trial registration: The trial is registered at www.clincaltrials.gov with the number NCT02879656, registration date 08/17/2016.

Protocol version: This document has been written based on the information contained in the trial protocol published in BMC Musculoskeletal Disorders [1].

Statistical Analysis Plan (SAP) revision history:

SAP version	Section changed	Description and reason for change	Date changed
1.0	Initial draft		
1.1	References	References added	13.5.2024

Roles and responsibilities:

Role	Name
SAP author	Teemu Hevonkorpi, Antti Launonen
Study trialist	Aleksi Reito
Senior statistician	Sakke Purolainen
Chief investigator	Teemu Hevonkorpi

Signatures:

Role	Name	Date	Signature
SAP author	Antti Launonen	13.5.2024	
Senior statistician	Sakke Purolainen	13.5.2024	
Study trialist	Aleksi Reito	13.5.2024	

Chief investigator	Teemu Hevonkorpi	13.5.2024	

SECTION 1: INTRODUCTION

1.1 Background

In the aging population, distal radius fracture (DRF) is one of the most common fractures and accounts for 4% of all fractures [2]. The age-adjusted overall incidence of DRF has varied between 100 to 300 per 100,000 person-years depending on sample, and it is more common (200 to 1200 per 100,000 person-years) in the elderly population [3,45,6]. In Finland, the estimated annual number of DRFs among patients aged 60 and older is approximately 8000 to 9000.

In addition to the significant disability caused by DRFs among older individuals, these injuries are associated with a high economic impact. In general, the operative interventions, outpatient visits, and rehabilitation after a fracture put additional strains on scarce resources. The minimum direct cost of every operatively-treated fracture has been estimated to be approximately 1400 to 6800 € [5,6]. Considering the annual number of these fractures, it is essential that our limited resources are targeted at treatment methods with proven efficacy and cost-effectiveness.

Treatment options for DRFs have varied between nonoperative and operative, and numerous different surgical methods have been described over time. Operative treatment with a volar locking plate was introduced in the early 2000s, and since then the procedure rapidly gained its place as the most frequent fixation method of DRF [7,8].

It has been shown that the functional outcome of a DRF correlates with the anatomical position of the articular surface in young, active patients. In the older population, however, the anatomic parameters of the DRF correlate poorly with a positive long-term functional outcome [9–11] and the issue whether or not to operate these fractures has been under a discussion among the orthopaedic and hand surgeons over the past few years.

The aim of this randomized prospective trial will be to compare the non-operative and operative treatment of initially or early malaligned distal radius fractures in patients aged 65 and older in terms of functional outcome.

SECTION 2: STUDY METHODS

2.1 Trial Design

This is a prospective, superiority, randomized, controlled, multicenter and multinational trial that compares the non-operative and operative treatment of initially or early malaligned distal radius fractures in patients aged 65 and older.

The Statistical Analysis Plan (SAP) is reported in accordance with the "Guidelines for the Content of Statistical Analysis Plan in Clinical Trials".

The DRIFT trial is registered at www.clincaltrials.gov with the number NCT02879656, registration date 08/17/2016. The primary ethical approval for the trial was given by the medical ethics committee of Tampere University Hospital (Regional Ethics Committee of Tampere University Hospital, ETL-code
R16105).

All sites gained institutional approval before the start of the trial. Every patient included in the trial and those patients who declined but were asked to take part in the external follow-up cohort were asked to give written informed consent.

2.2 Randomization

Patients were randomized in block allocation fashion. The blocks were stratified by age (65–75 years and >75 years) and intra vs. extra-articular fracture. The treatment allocations from the matrix were acquired from an online randomization system (website http:// randomize. net), where the researcher logged in after written consent and received the correct intervention. The physician responsible for the intervention or treatment did not participate in any part of the collection of patient outcomes during the follow-ups.

2.3 Sample Size

Assuming the effect size of a 11-point difference (previously reported MCID) [12] in the PRWE score and an SD of 14 points. Based on power calculations (CI 95%, power 0.95, SD 14), the required sample size per group was 40 patients. Assuming a 30% drop-out rate based on possible surgical intervention during cast treatment, group size needed was determined to be 57 per group (total 114). When two cohorts are taken into account, a total of 228 patients were needed.

2.4 Framework

The overall objective of the trial was to determine whether operative treatment of the early or late malaligned DRF with volar locked plating (VLP) yield in clinically and statistically superior results when compared to non-operative treatment. The primary outcome in this study was the patient rated wrist evaluation (PRWE) score measured after 1 year.

2.5 Timing of the analysis

The analysis for the primary outcome PRWE will be performed after the last follow-up assessment at 1 year. The main publication of the trial will be prepared when these data are available.
In addition, an additional analysis will be performed when 2-years follow-up assessments are available.

2.6 Timing of outcome assessments

The trial consists of the following time points; baseline, 5 to 10 days (arms 3N, 3O and 4), 5 to 6 weeks, 3 months, 1 year and 2 years. An overview of the assessments and procedures has been presented in the protocol.

SECTION 3: STATISTICAL PRINCIPLES

3.1 Confidence intervals and P values

For the primary outcome PRWE the statistical tests will be two-sided and a p-value <0.05 will be considered statistically significant. Confidence intervals will be 95% (95% CI) and two-sided. For the secondary outcomes the use of p values will be toned down when interpreting the results.

3.2 Analysis populations

The primary analysis will be performed using the Intention to Treat (ITT) principle. Patients allocated to either operative or non-operative group treatment are followed up, assessed and analyzed as members of that group, regardless of their adherence to the planned course of treatment.

SECTION 4: TRIAL POPULATION

4.1 Screening data

At all participating hospitals, patients eligible for treatment were screened from the digital imaging software PACS for the radiologic inclusion and exclusion criteria. If they fulfilled the inclusion criteria, electronic patient records were checked over for other possible exclusion criteria. If the patient fulfilled all the inclusion criteria, they were invited to join the study. The number of patients who did not meet the criteria and the reason for ineligibility will be reported in a CONSORT flow chart.

4.2 Eligibility

The following criteria was used throughout the study for patient selection.

Inclusion criteria:

- low energy intra or extra-articular dorsally displaced distal radius fracture within 3 cm of the radiocarpal joint, diagnosed with lateral and posterior-anterior radiographs in ER
- >10° dorsal tilt and/or over 2 mm step-off and/or over 3 mm shortening in the radiographs

Exclusion criteria:

- Refusal to participate in the study
- Open fracture more than Gustilo 1 gradus
- Patient aged under 65
- Chauffeure's or Barton´s fracture
- Smith's fracture
- Does not understand written and spoken guidance in local languages
- Pathological fracture or previous fracture in the same wrist or forearm

4.3 Recruitment

The CONSORT flowchart will present the number of patients screened, excluded (with reasons), eligible for inclusion, randomized, receiving allocated treatment, withdrawals (with reasons), lost to follow-up (with reasons), included in the ITT analysis, included in the per protocol analysis.

4.4 Withdrawal/follow-up

The included patients were allowed to withdraw from the study at any time during the study period. In case a patient decided to withdraw from the study, the data before the withdrawal was used normally in the analyses.

4.5 Baseline patient characteristics

Baseline characteristics will be presented. Categorical variables will be presented as numbers and percentages. Continuous variables will be presented as mean with standard deviation (SD), if normally distributed and as median with interquartile range (IQR) if skewedly distributed. Imbalances between the treatment groups will be noted. Baseline and follow-up values for the primary and secondary outcomes will be presented as part of the analysis.

SECTION 5: ANALYSIS

5.1 Outcome definitions

5.1.1 Primary outcome

The primary outcome measure will be the Patient-Reported Wrist Evaluation (PRWE) score at 1 year follow-up. The PRWE has 15 questions with regard to the subjective function and pain of the wrist and hand rated on an 11-point scale from 0 to 10, giving a total range of 0 to 100 (in which 0 is the best). The minimal clinically important difference (MCID) for the PRWE has been reported to be 11 points.

5.1.2 Key secondary outcomes

The key secondary outcomes measured will be QuickDASH (Disabilities of Arm, Shoulder and Hand), PCS (pain catastrophizing scale), VAS, grip strength, health-related quality of life (15-D), complications, and the number of surgical interventions in the non-operatively treated group at 1 year follow-up.

5.1.3 Secondary outcomes

The secondary outcomes measured will be physical activity and the number of wrist movements measured with Axivity accelerometer and clinical frailty (frailty score). EWC(Edinburgh Wrist Calculator) will be used to assess the probability of radiological malalignment from initial radiographs after injury.

SECTION 6: ANALYSIS METHODS

6.1 Primary analysis method

The primary analysis method for PRWE will be a linear mixed model. The patient is included as a random factor. Study group and follow-up assessments (3 months, 1 year, 2 years) will be included as a fixed factor. Study center and patient age will also be included as fixed factors. The main model will include the interaction between the study group and the follow-up assessment. Primary treatment effect at 1 year will be estimated as the estimated marginal means between the study group. Similar estimates will be obtained for 3 months and later 2 years also. Similar analysis will be done for key secondary outcomes QuickDASH, VAS pain, grip strength and 15-D. Results will be presented with 95% CIs.

For categorical variables we will estimate the group absolute risk difference using a logistic regression. Separate models will be run at each follow-up time-points. Logistic models include study group, study center and patient age as covariates.

6.2 Missing data

As stated above, imputations will not be applied in this study due to the repeated mixed model analysis. Each randomized patient will be included in the intention-to-treat analysis with the collected data.

6.3 Additional analyses

For primary and secondary outcomes an additional analysis will be done with a crude between-group comparison. For continuous variables we will assess the study group difference using Welch t-test at each time point. Categorical variables will be compared using Fisher exact test for 2x2 tables and Chi-squares test for other comparisons.

The subgroup analyses for the primary outcome will be done according to following variables: of age (per stratification), sex, fracture group (intra vs. extra-articular) and smoking. These variables will be evaluated against the PRWE and QuickDASH

scores and overall quality of life after fracture. The analysis will be performed with linear mixed model.

Sensitivity and exploratory analyses will be performed with the purpose of testing the robustness of the intention-to-treat analysis, including a per-protocol analysis for the primary outcome; Crossover patients are defined as patients allocated to the non-operative group who undergo operative treatment with VLP during follow-up.

Additionally, we will make a variety of radiological analyses aiming to assess the association between x-ray parameters and the PROM's. EWC(Edinburgh Wrist Calculator) will be used to assess the probability of radiological malalignment from initial radiographs after injury.

Analysis on cost-effectiviness sand differences in treatment costs between the treatment groups will be performed.

The number and intensity of wrist movements recorded with the Axivity accelerometer will be analyzed. Comparison between treatment groups and the association between the wrist movements and PROM's will be evaluated.

6.4 Harms

Adverse and serious adverse events will be presented as number and percentage for each event.

Adverse events (AE) were classified as follows:

- Superficial wound infection
- Carpal tunnel syndrome
- Other neuropathies or nerve injury
- Tendon laceration / rupture
- Fracture nonunion at 3 months
- Symptomatic malunion
- Implant failure
- Poor active range of motion or finger stiffness affecting daily living at 12 months
- CRPS

- Persistent pain (other than CRPS) over 3 months requiring opiate-level medication

Adverse event was considered to be serious (SAE) if it resulted in inpatients hospitalization, prolonged hospitalization, life-threatening condition or death of the patient:

- Deep wound infection / infection requiring i.v. antibiotics
- Acute cardiopulmonary conditions (e.g. pulmonary embolism, myocardial infarction, acute heart failure)
- Single / multi-organ dysfunction
- Death

6.5 Statistical software

All statistical analysis will be made using the latest R software.

SECTION 7: REFERENCES

1. Hevonkorpi TP, Launonen AP, Raittio L, et al. Nordic Innovative Trial to Evaluate OsteoPorotic Fractures (NITEP-group): non-operative treatment versus surgery with volar locking plate in the treatment of distal radius fracture in patients aged 65 and over - a study protocol for a prospective, randomized controlled trial. *BMC Musculoskelet Disord*. 2018;19(1):106. doi:10.1186/s12891-018-2019-5
2. Court-Brown CM, Caesar B. Epidemiology of adult fractures: A review. *Injury*. 2006;37(8):691-697. doi:10.1016/j.injury.2006.04.130
3. Flinkkilä T, Sirniö K, Hippi M, et al. Epidemiology and seasonal variation of distal radius fractures in Oulu, Finland. *Osteoporos Int*. 2011;22(8):2307-2312. doi:10.1007/s00198-010-1463-3
4. Brogren E, Petranek M, Atroshi I. Incidence and characteristics of distal radius fractures in a southern Swedish region. *BMC Musculoskelet Disord*. 2007;8(1):48. doi:10.1186/1471-2474-8-48
5. Shauver MJ, Clapham PJ, Chung KC. An economic analysis of outcomes and complications of treating distal radius fractures in the elderly. *Journal of Hand Surgery*. 2011;36(12). doi:10.1016/j.jhsa.2011.09.039
6. Pirkanmaan sairaanhoitopiiri. Tuotehinnasto. 2017.
7. Hevonkorpi TP, Launonen AP, Huttunen TT, Kannus P, Niemi S, Mattila VM. Incidence of distal radius fracture surgery in Finns aged 50 years or more between 1998 and 2016 – too many patients are yet operated on? *BMC Musculoskelet Disord*. 2018;19(1):70. doi:10.1186/s12891-018-1983-0
8. Mattila VM, Huttunen TT, Sillanpää P, Niemi S, Pihlajamäki H, Kannus P. Significant Change in the Surgical Treatment of Distal Radius Fractures: A Nationwide Study Between 1998 and 2008 in Finland. *The Journal of Trauma: Injury, Infection, and Critical Care*. 2011;71(4):939-943. doi:10.1097/TA.0b013e3182231af9
9. Leung F, Ozkan M, Chow SP. Conservative treatment of intra-articular fractures of the distal radius--factors affecting functional outcome. *Hand Surg*. 2000;5(2):145-153. Accessed April 18, 2018. http://www.ncbi.nlm.nih.gov/pubmed/11301509

10. Luokkala T, Laitinen M, Hevonkorpi T, Raittio L, Mattila V, Launonen A. Distal radius fractures in the elderly population. *EFORT Open Rev*. 2020;Jun 17;5(6.
11. Plant CE, Parsons NR, Costa ML. Do radiological and functional outcomes correlate for fractures of the distal radius? *Bone and Joint Journal*. 2017;99B(3):376-382. doi:10.1302/0301-620X.99B3.35819/LETTERTOEDITOR
12. Walenkamp MMJ, de Muinck Keizer RJ, Goslings JC, Vos LM, Rosenwasser MP, Schep NWL. The Minimum Clinically Important Difference of the Patient-rated Wrist Evaluation Score for Patients With Distal Radius Fractures. *Clin Orthop Relat Res*. 2015;473(10):3235-3241. doi:http://dx.doi.org/10.1007/s11999-015-4376-9
