# Supplementary material for: Nonoperative treatment versus volar locking plating for distal radius fracture in patients aged 65 years or older (DRIFT trial): A randomized controlled trial
Source: PLoS Med. 2025 Sep 5;22(9):e1004728. doi: 10.1371/journal.pmed.1004728 (PMC12425212; doi:10.1371/journal.pmed.1004728)
Supplement: S3 Text — (DOCX) [file pmed.1004728.s005.docx]

**DRIFT TRIAL - Adverse events**

At the 12-month follow-up, we found 25 treatment-related adverse events and 11 other adverse events (AE).

Primarily malaligned DRF

10/66 (15%) patients treated non-operatively and 5/58 (9%) operated with VLP had a complication. In the non-operative treatment group, there were six carpal tunnel syndromes leading to operative release, one pressure ulcer caused by the cast, and three symptomatic malunions. In the operative treatment group, there were two carpal tunnel syndromes requiring neurolysis, one plate loosening proximally leading to re-operation and two VLP’s with distal screw loosening leading to removal of the plate after fracture union.

Early malaligned DRF

2/44 (5%) patients treated non-operatively and 3/42 (7%) patients operated with VLP had a complication. In the non-operative treatment group, there was one symptomatic malunion leading to corrective osteotomy eight months after the fracture and one complex regional pain syndrome (CRPS). In the operative treatment group, one carpal tunnel syndrome required neurolysis, one superficial wound infection required peroral antibiotics, and one suture material fistula that healed without any treatment.

Well-aligned DRF

In patients with satisfactory fracture alignment in the first follow-up visit, 5/62 (8%) had a complication. There was one extensor pollicis longus (EPL) tendon rupture, one extensor digitorum communis (EDC) dysfunction requiring extra rehabilitation, one non-union leading to operative treatment with bone transfer, and two symptomatic malunions treated non-operatively due to the patient’s preferences.

**Adverse events at 12-months follow-up by study group**

| **Treatment groups** | **Arm 1 (n=66) (Primarily malaligned DRF, Non-Operative)** | **Arm 2 (n=58) (Primarily malaligned DRF, Operative)** | **Arm 3N (n=44) (Early malaligned DRF, Non-Operative)** | **Arm 3O (n=42) (Early malaligned DRF, Operative)** | **Arm 4 (n=63) (Well-aligned DRF,  Non-Operative)** | **External Group (n=18)** |
| --- | --- | --- | --- | --- | --- | --- |
| **Treatment related adverse-events** |  |  |  |  |  |  |
| Carpal tunnel syndrome | 6/66 (9%) | 2/58 (3%) | 0 | 1/42 (2%) | 0 | 0 |
| Symptomatic malunion | 3/66 (3%) | 0 | 1/44 (2%) | 0 | 2/63 (3%) | 0 |
| Nonunion | 0 | 0 | 0 | 0 | 1/63 (2%) | 0 |
| Plate/screw loosening | 0 | 3/58 (5%) | 0 | 0 | 0 | 0 |
| CRPS | 0 | 0 | 1/44 (2%) | 0 | 0 | 0 |
| Superficial infection | 0 | 0 | 0 | 2/42 (5%) | 0 | 0 |
| Deep infection | 0 | 0 | 0 | 0 | 0 | 0 |
| Tendon rupture/ dysfunction | 0 | 0 | 0 | 0 | 2/63 (3%) | 0 |
| Pressure ulcer | 1/66 (2%) | 0 | 0 | 0 | 0 | 0 |
| **Adverse events unrelated to received treatment** |  |  |  |  |  |  |
| Acute myocardial infarction | 1/66 (2%) | 0 | 0 | 0 | 0 | 0 |
| Cerebral infarction / TIA | 1/66 (2%) | 1/58 (2%) | 0 | 0 | 0 | 0 |
| Other hospitalized infection | 0 | 1/58 (2%) | 0 | 1/42 (2%) | 0 | 0 |
| Other fracture | 2/66 (3%) | 0 | 2/44 (4.5%) | 0 | 1/63 (2%) | 0 |
| Death | 0 | 1/58 (2%) | 0 | 0 | 0 | 0 |

Data are presented as number (percentage)
CRPS = complex regional pain syndrome , DRF=distal radius fracture, TIA = transient ischemic attack

**Clavien-dindo classification of post-operative complications**

| **Treatment groups** | **Arm 2 (n=58) (Primarily malaligned DRF, Operative)** | **Arm 3O (n=42) (Early malaligned DRF, Operative)** |
| --- | --- | --- |
| Grade I | 0 | 1 (2%) |
| Grade II | 0 | 1 (2%) |
| Grade IIIa | 5 (9%) | 1 (2%) |
| Grade IIIb | 0 | 0 |
| Grade IV | 0 | 0 |
| Grade V | 0 | 0 |

Clavien-Dindo classification[1] of operatively treated patients of the DRIFT trial.
Grade I: Minor deviations from the normal postoperative course, such as minor wound infection treated with local measures or mild nausea.

Grade II: Complications requiring pharmacological treatment (e.g., antibiotics, diuretics) or blood transfusions.

Grade III: Complications requiring invasive intervention:

IIIa: Intervention performed without general anesthesia.

IIIb: Intervention performed under general anesthesia or endoscopic or radiological intervention.

Grade IV: Life-threatening complications requiring ICU management:

IVa: Single organ dysfunction (e.g., respiratory failure, renal failure).

IVb: Multi-organ dysfunction.

Grade V: Death.

DRF=distal radius fracture

1. Dindo D, Demartines N, Clavien PA. Classification of Surgical Complications: A New Proposal With Evaluation in a Cohort of 6336 Patients and Results of a Survey. Ann Surg. 2004;240: 205. doi:10.1097/01.SLA.0000133083.54934.AE
