# Supplementary material for: Nonoperative treatment versus volar locking plating for distal radius fracture in patients aged 65 years or older (DRIFT trial): A randomized controlled trial
Source: PLoS Med. 2025 Sep 5;22(9):e1004728. doi: 10.1371/journal.pmed.1004728 (PMC12425212; doi:10.1371/journal.pmed.1004728)
Supplement: S4 Text — (DOCX) [file pmed.1004728.s006.docx]

**DRIFT TRIAL - Deviations from the original study protocol**

A few changes occurred to the original protocol ^1^ during the trial. These changes and clarifications are explained below.

**Participating centers**

In addition to participating hospitals stated in the original manuscript of the study protocol (Tampere University Hospital, Central Finland Central Hospital (current name Hospital Nova), Satakunta Central Hospital and Viborg regional Hospital), department of orthopaedics of Karolinska University Hospital (Huddinge, Stockholm, Sweden) was willing to join the trial and started in 1/2020.

**Final group sizes**

Based on power calculations (Cl 95%, power 0.95), with an assumption of the effect size of an 11-point difference (previously reported MCID of the PRWE score) in the PRWE score and an SD of 14 points, the required sample size per Arm was 40 patients. Assuming a 30% drop-out rate, the group size needed was determined as 57 per Arm.

Recruitment of primarily malaligned DRFs to Arm1 and Arm2 of the trial was quicker than recruitment of early malaligned DRFs to Arm3N and Arm3O. Nine extra patients were recruited to Arm 1 and one extra patient was recruited to Arm 2 before the recruitment was ended in all participating centers. In patients with early malaligned DRF, we had to cease the recruitment of patients before reaching 57 patients per Arm due to the decreased inclusion rate and changed treatment policies towards more non-operative treatment. The decision to cease the recruitment was done by the entire research group. We managed to recruit 44 patients to Arm3N and 42 patients to Arm3O. As the drop-out rate was much lower than expected, the 12-months analysis included at minimum 40 patients in each Arm, as was originally intended.

**Recruiting time**

The recruiting time was expected to be 2 years. However, during the recruitment period we faced unexpected adversities (COVID-19 pandemic 3/2020 – 6/2022, Finnish nurses’ strike 4/2022 – 8/2022), which caused difficulties to conduct the recruitment of the trial between 2020 and 2023. At finally, the recruitment time period was 5 years and 4 months (2/2018 to 6/2023).

**Liner mixed model**

Deviating from the published study protocol, a linear mixed model was chosen as the primary analysis method as it is currently the most common method in longitudinal trials with repeated measurement setup.

**Analysis of accelerometer data (Axivity) and Edinburgh Wrist Calculator (EWC)**

The current publication focuses on primary outcome (PRWE) and key secondary outcomes (QuickDASH, VAS, grip strength, health-related quality of life (15-D), complications, and the number of concomitant surgeries). The analysis of other secondary outcomes will be done in the future and the results published as secondary analysis of the trial.

1. Hevonkorpi TP, Launonen AP, Raittio L, et al. Nordic Innovative Trial to Evaluate OsteoPorotic Fractures (NITEP-group): non-operative treatment versus surgery with volar locking plate in the treatment of distal radius fracture in patients aged 65 and over - a study protocol for a prospective, randomized controlled trial. BMC Musculoskelet Disord. 2018;19(1):106. doi:10.1186/s12891-018-2019-5
