# Supplementary material for: Nonoperative treatment versus volar locking plating for distal radius fracture in patients aged 65 years or older (DRIFT trial): A randomized controlled trial
Source: PLoS Med. 2025 Sep 5;22(9):e1004728. doi: 10.1371/journal.pmed.1004728 (PMC12425212; doi:10.1371/journal.pmed.1004728)
Supplement: S5 Text — (DOCX) [file pmed.1004728.s007.docx]

**DRIFT TRIAL – Additional analyses**

**Per protocol analysis**Per protocol analysis of primarily malaligned distal radius fractures

confint(emmeans(prwe12,pairwise~perp|time,lmer.df = "satterthwaite"))

## $emmeans

## time = 3 months:

## perp emmean SE df lower.CL upper.CL

## Nonoperative 25.40 2.90 163 19.67 31.1

## VLP 18.60 3.03 163 12.61 24.6

##

## time = 12 months:

## perp emmean SE df lower.CL upper.CL

## Nonoperative 18.59 2.84 158 12.98 24.2

## VLP 8.87 2.94 154 3.07 14.7

##

## Results are averaged over the levels of: strata1, strata2

## Degrees-of-freedom method: satterthwaite

## Confidence level used: 0.95

##

## $contrasts

## time = 3 months:

## contrast estimate SE df lower.CL upper.CL

## Nonoperative - VLP 6.81 4.11 165 -1.30 14.9

##

## time = 12 months:

## contrast estimate SE df lower.CL upper.CL

## Nonoperative - VLP 9.72 3.99 158 1.84 17.6

##

## Results are averaged over the levels of: strata1, strata2

## Degrees-of-freedom method: satterthwaite

## Confidence level used: 0.95

Per protocol analysis of early malaligned distal radius fractures

confint(emmeans(prwe34,pairwise~perp|time,lmer.df = "satterthwaite"))

## $emmeans

## time = 3 months:

## perp emmean SE df lower.CL upper.CL

## Nonoperative 22.78 3.11 90.2 16.60 29.0

## VLP 17.55 3.39 90.6 10.82 24.3

##

## time = 12 months:

## perp emmean SE df lower.CL upper.CL

## Nonoperative 16.53 3.23 97.9 10.13 22.9

## VLP 9.54 3.43 93.3 2.72 16.4

##

## Results are averaged over the levels of: strata1, strata2

## Degrees-of-freedom method: satterthwaite

## Confidence level used: 0.95

##

## $contrasts

## time = 3 months:

## contrast estimate SE df lower.CL upper.CL

## Nonoperative - VLP 5.23 4.5 91.9 -3.72 14.2

##

## time = 12 months:

## contrast estimate SE df lower.CL upper.CL

## Nonoperative - VLP 7.00 4.6 96.2 -2.13 16.1

##

## Results are averaged over the levels of: strata1, strata2

## Degrees-of-freedom method: satterthwaite

## Confidence level used: 0.95

**Subgroup analysis by age**

Subgroup analysis of Patient-Rated Wrist Evaluation at 3-month and 12-month follow-up in patients aged 65 to 74 years with primarily malaligned distal radius fracture

confint(emmeans(prwe12strata2alle,pairwise~study_arm|time,lmer.df = "satterthwaite"))

## $emmeans

## time = time03:

## study_arm emmean SE df lower.CL upper.CL

## Arm1 25.03 3.86 110 17.38 32.7

## Arm2 19.55 3.72 107 12.17 26.9

##

## time = time12:

## study_arm emmean SE df lower.CL upper.CL

## Arm1 19.64 3.71 104 12.28 27.0

## Arm2 9.81 3.67 105 2.52 17.1

##

## Results are averaged over the levels of: strata1

## Degrees-of-freedom method: satterthwaite

## Confidence level used: 0.95

##

## $contrasts

## time = time03:

## contrast estimate SE df lower.CL upper.CL

## Arm1 - Arm2 5.47 5.31 109 -5.058 16.0

##

## time = time12:

## contrast estimate SE df lower.CL upper.CL

## Arm1 - Arm2 9.83 5.18 106 -0.438 20.1

##

## Results are averaged over the levels of: strata1

## Degrees-of-freedom method: satterthwaite

## Confidence level used: 0.95

Arm1=non-operative, Arm2= volar locking plating


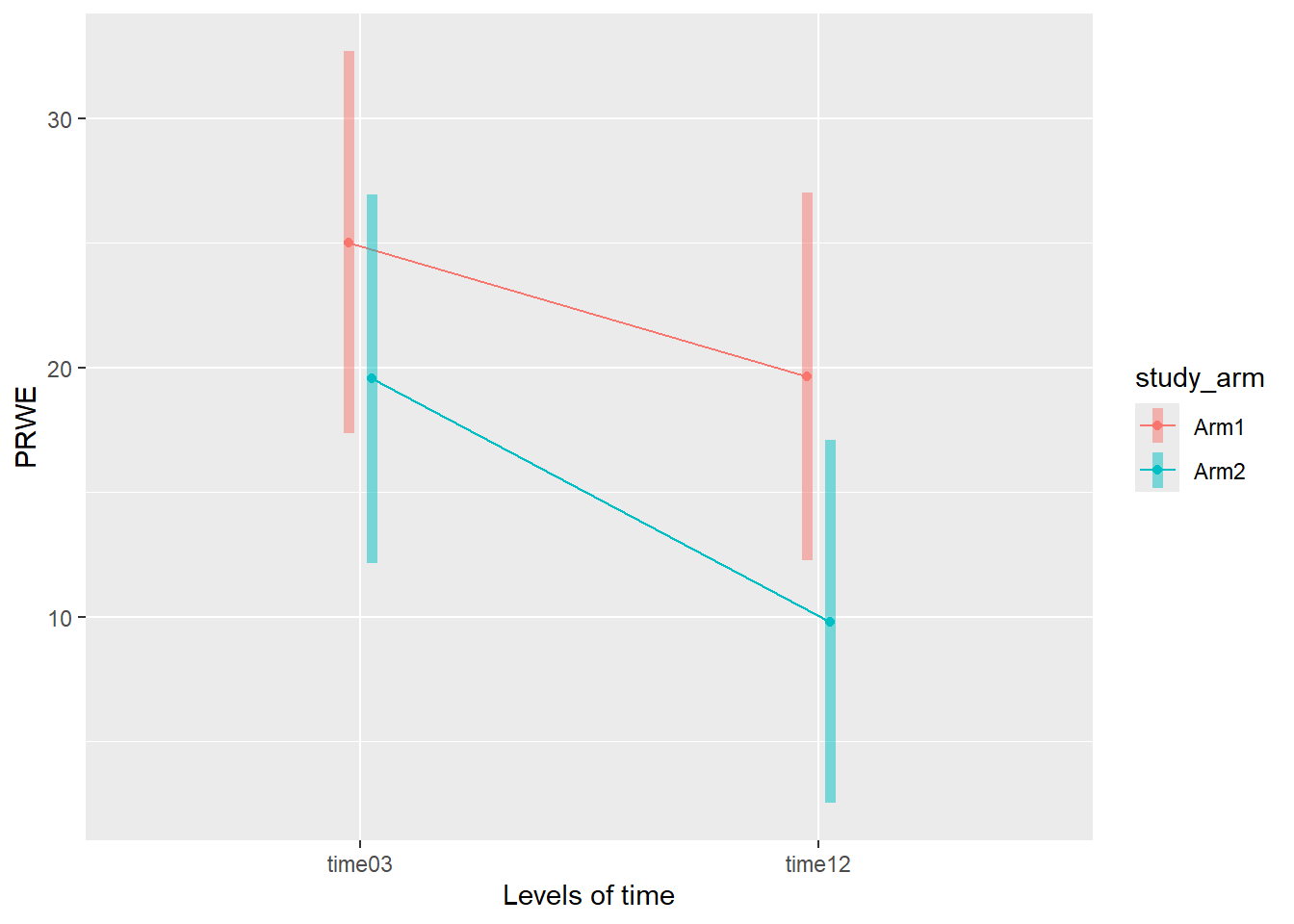


Subgroup analysis of Patient-Rated Wrist Evaluation (PRWE) at 3-month and 12-month follow-up in patients aged 65 to 74 years with primarily malaligned distal radius fracture. Arm1=non-operative, Arm2= volar locking plating

**Subgroup analysis of Patient-Rated Wrist Evaluation at 3-month and 12-month follow-up in patients aged 75 years or older with primarily malaligned distal radius fracture**

confint(emmeans(prwe12strata2yli,pairwise~study_arm|time,lmer.df = "satterthwaite"))

## $emmeans

## time = time03:

## study_arm emmean SE df lower.CL upper.CL

## Arm1 26.6 4.14 48.8 18.24 34.9

## Arm2 16.1 4.94 52.7 6.17 26.0

##

## time = time12:

## study_arm emmean SE df lower.CL upper.CL

## Arm1 16.9 4.13 48.5 8.65 25.2

## Arm2 7.6 4.70 46.9 -1.85 17.1

##

## Results are averaged over the levels of: strata1

## Degrees-of-freedom method: satterthwaite

## Confidence level used: 0.95

##

## $contrasts

## time = time03:

## contrast estimate SE df lower.CL upper.CL

## Arm1 - Arm2 10.47 6.41 51.5 -2.38 23.3

##

## time = time12:

## contrast estimate SE df lower.CL upper.CL

## Arm1 - Arm2 9.34 6.21 47.8 -3.14 21.8

##

## Results are averaged over the levels of: strata1

## Degrees-of-freedom method: satterthwaite

## Confidence level used: 0.95

Arm1=non-operative, Arm2= volar locking plating


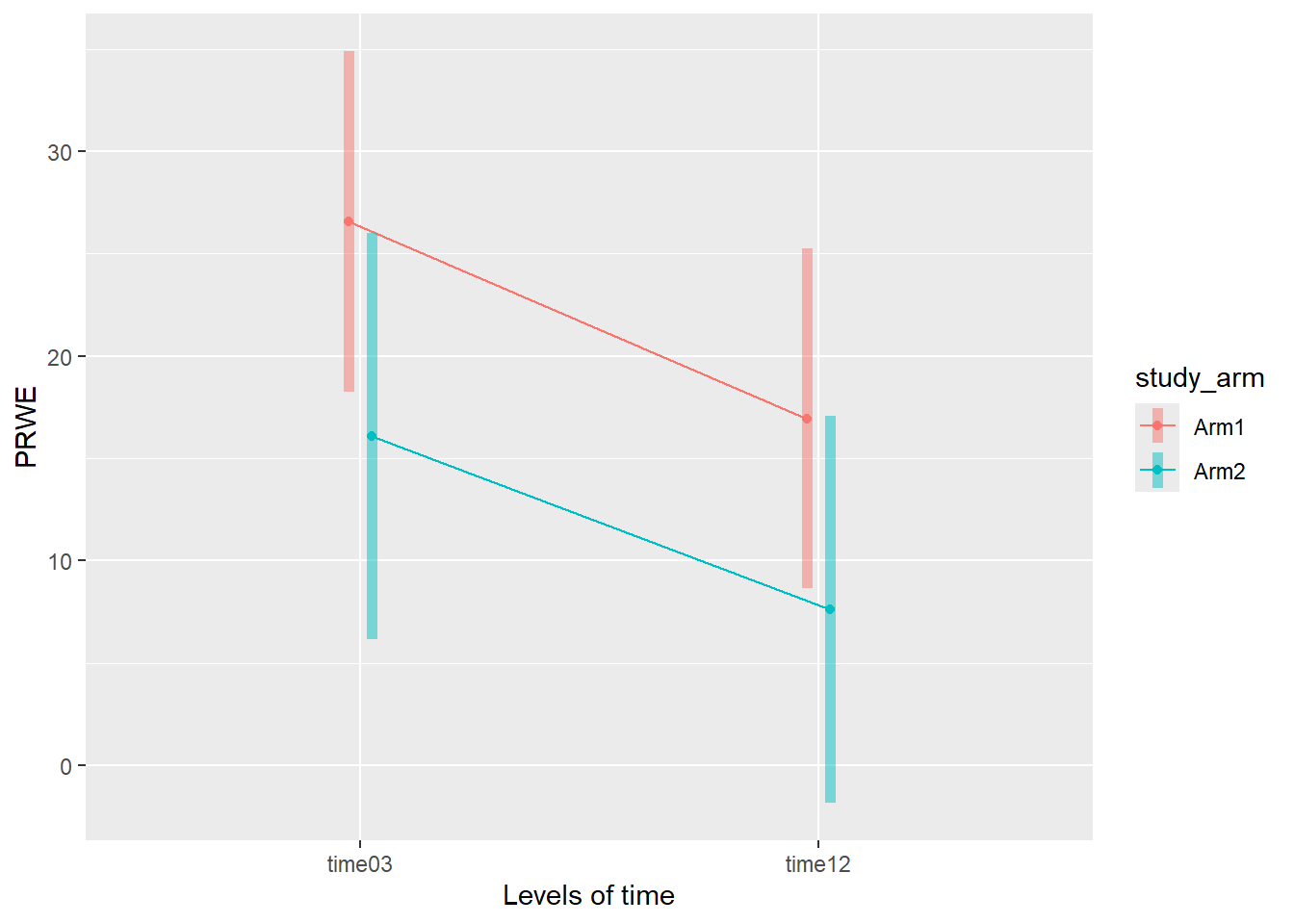


Subgroup analysis of Patient-Rated Wrist Evaluation at 3-month and 12-month follow-up in patients aged 75 years or older with primarily malaligned distal radius fracture. Arm1=non-operative, Arm2= volar locking plating

Subgroup analysis of Patient-Rated Wrist Evaluation at 3-month and 12-month follow-up in patients aged 65 to 74 years with early malaligned (5 to 10 days) distal radius fracture

confint(emmeans(prwe34strata2alle,pairwise~study_arm|time,lmer.df = "satterthwaite"))

## $emmeans

## time = time03:

## study_arm emmean SE df lower.CL upper.CL

## Arm3N 24.9 3.79 64.9 17.37 32.5

## Arm3O 16.7 4.39 65.9 7.90 25.4

##

## time = time12:

## study_arm emmean SE df lower.CL upper.CL

## Arm3N 18.2 3.91 68.3 10.44 26.0

## Arm3O 10.1 4.41 66.3 1.32 18.9

##

## Results are averaged over the levels of: strata1

## Degrees-of-freedom method: satterthwaite

## Confidence level used: 0.95

##

## $contrasts

## time = time03:

## contrast estimate SE df lower.CL upper.CL

## Arm3N - Arm3O 8.28 5.75 66.2 -3.21 19.8

##

## time = time12:

## contrast estimate SE df lower.CL upper.CL

## Arm3N - Arm3O 8.13 5.82 67.6 -3.50 19.7

##

## Results are averaged over the levels of: strata1

## Degrees-of-freedom method: satterthwaite

## Confidence level used: 0.95

Arm3N=non-operative, Arm3O= volar locking plating


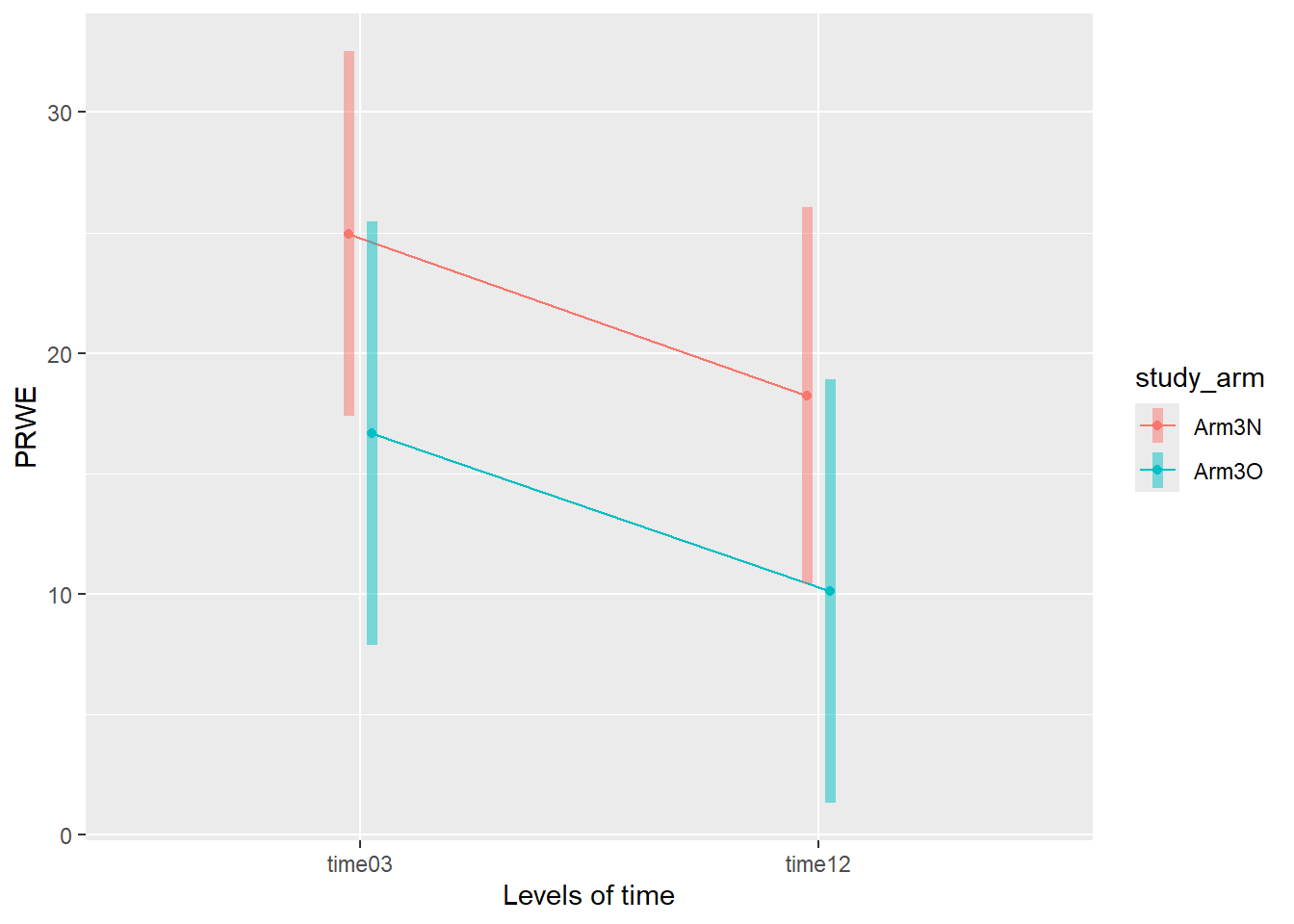


Subgroup analysis of Patient-Rated Wrist Evaluation at 3-month and 12-month follow-up in patients aged 65 to 74 years with early malaligned (5 to 10 days) distal radius fracture. Arm3N=non-operative, Arm3O= volar locking plating

Subgroup analysis of Patient-Rated Wrist Evaluation at 3-month and 12-month follow-up in patients aged 75 years or older with early malaligned (5 to 10 days) distal radius fracture.

confint(emmeans(prwe34strata2yli,pairwise~study_arm|time,lmer.df = "satterthwaite"))

## $emmeans

## time = time03:

## study_arm emmean SE df lower.CL upper.CL

## Arm3N 17.0 5.37 23.1 5.893 28.1

## Arm3O 21.0 4.89 22.5 10.829 31.1

##

## time = time12:

## study_arm emmean SE df lower.CL upper.CL

## Arm3N 13.2 5.44 24.1 1.981 24.4

## Arm3O 11.1 4.96 23.6 0.858 21.4

##

## Results are averaged over the levels of: strata1

## Degrees-of-freedom method: satterthwaite

## Confidence level used: 0.95

##

## $contrasts

## time = time03:

## contrast estimate SE df lower.CL upper.CL

## Arm3N - Arm3O -3.95 7.37 22.7 -19.2 11.3

##

## time = time12:

## contrast estimate SE df lower.CL upper.CL

## Arm3N - Arm3O 2.10 7.47 23.8 -13.3 17.5

##

## Results are averaged over the levels of: strata1

## Degrees-of-freedom method: satterthwaite

## Confidence level used: 0.95

Arm3N=non-operative, Arm3O= volar locking plating


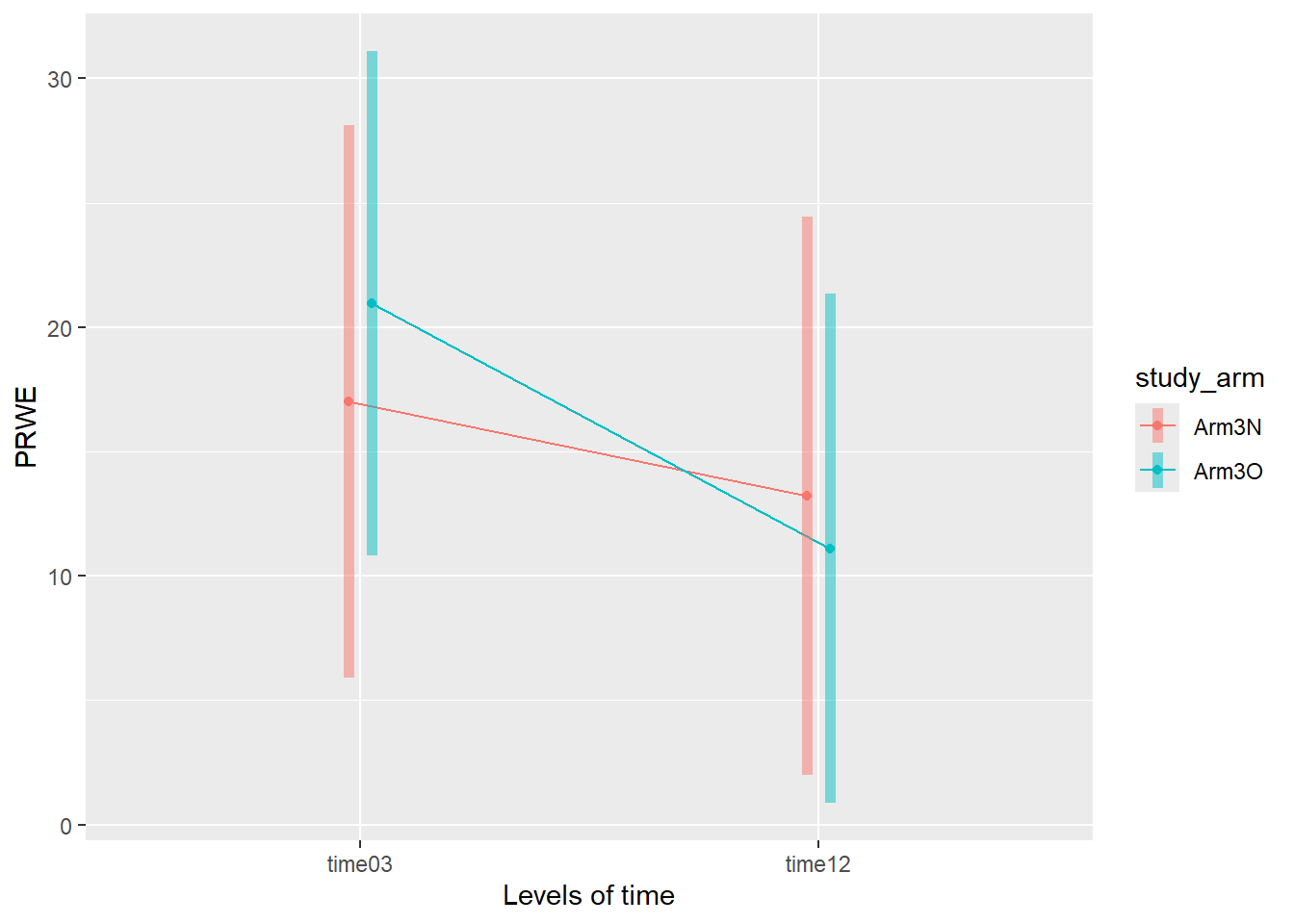


Subgroup analysis of Patient-Rated Wrist Evaluation at 3-month and 12-month follow-up in patients aged 75 years or older with early malaligned (5 to 10 days) distal radius fracture. Arm3N=non-operative, Arm3O= volar locking plating
