# Supplementary material for: Nonoperative treatment versus volar locking plating for distal radius fracture in patients aged 65 years or older (DRIFT trial): A randomized controlled trial
Source: PLoS Med. 2025 Sep 5;22(9):e1004728. doi: 10.1371/journal.pmed.1004728 (PMC12425212; doi:10.1371/journal.pmed.1004728)
Supplement: S6 Text — (DOCX) [file pmed.1004728.s008.docx]

**DRIFT TRIAL – Patient involvement in the trial**

To improve patient involvement in this trial, we interviewed patients with DRF before the onset of the trial. The aim of the interviews was to move towards more patient-centered medicine by taking into account the patients’ preferences and beliefs for a good outcome. We asked the same questions at the

beginning of the treatment and at the 12-month follow-up. The questionnaires used for patient self-assessment are attached in this supplement file. The analysis of the patient self-assessment questionnaires will be published in a separate publication with other additional data analysis.

**Patient self-assessment – initial situation Date: 21.5.2017 (ver 1.0) Researcher: Antti Launonen**

**Answer the following statements based on your expectations. Circle the most appropriate answer/number.**

|  | Completely disagree | Disagree | Neither agree or disagree | Agree | Completely agree |
| --- | --- | --- | --- | --- | --- |
| It is important for me that there is no pain in the wrist after the treatment has ended. | 1 | 2 | 3 | 4 | 5 |

|  | Completely disagree | Disagree | Neither agree or disagree | Agree | Completely agree |
| --- | --- | --- | --- | --- | --- |
| It is important for me that after the treatment my wrist operates the same way it did before the fracture. | 1 | 2 | 3 | 4 | 5 |

|  | Completely disagree | Disagree | Neither agree or disagree | Agree | Completely agree |
| --- | --- | --- | --- | --- | --- |
| It is important for me that after the treatment my wrist looks the same way it did before the fracture. | 1 | 2 | 3 | 4 | 5 |

|  | Completely disagree | Disagree | Neither agree or disagree | Agree | Completely agree |
| --- | --- | --- | --- | --- | --- |
| I believe that rehabilitation will have a more significant impact on the end result than whether I undergo surgery or not. | 1 | 2 | 3 | 4 | 5 |

|  | Completely disagree | Disagree | Neither agree or disagree | Agree | Completely agree |
| --- | --- | --- | --- | --- | --- |
| It is important for me to understand what is being done to me and why. | 1 | 2 | 3 | 4 | 5 |

**Patient self-assessment – 1 year, 2 years Date: 21.5.2017 (ver 1.0) Researcher: Antti Launonen**

**Answer the following statements and questions. Circle the most appropriate answer/number.**

|  | Completely disagree | Disagree | Neither agree or disagree | Agree | Completely agree |
| --- | --- | --- | --- | --- | --- |
| It is important for me that there is no pain in the wrist after the treatment has ended. | 1 | 2 | 3 | 4 | 5 |

|  | Completely disagree | Disagree | Neither agree or disagree | Agree | Completely agree |
| --- | --- | --- | --- | --- | --- |
| It is important for me that after the treatment my wrist operates the same way it did before the fracture. | 1 | 2 | 3 | 4 | 5 |

|  | Completely disagree | Disagree | Neither agree or disagree | Agree | Completely agree |
| --- | --- | --- | --- | --- | --- |
| It is important for me that after the treatment my wrist looks the same way it did before the fracture. | 1 | 2 | 3 | 4 | 5 |

|  | Completely disagree | Disagree | Neither agree or disagree | Agree | Completely agree |
| --- | --- | --- | --- | --- | --- |
| I believe that rehabilitation will have a more significant impact on the end result than whether I undergo surgery or not. | 1 | 2 | 3 | 4 | 5 |

|  | Completely disagree | | Disagree | Neither agree or disagree | Agree | Completely agree |
| --- | --- | --- | --- | --- | --- | --- |
| It is important for me to understand what is being done to me and why. | | 1 | 2 | 3 | 4 | 5 |

**1. Are you satisfied with the treatment you have received?**

**YES NO**

**2. Are you satisfied with how your wrist functions at the moment?**

**YES NO**

**3. Are you satisfied with participating in this study?**

**YES NO**

**4. Do you wish that chance had assigned you another treatment option?**

**YES NO**
