# Supplementary material for: Nonoperative treatment versus volar locking plating for distal radius fracture in patients aged 65 years or older (DRIFT trial): A randomized controlled trial
Source: PLoS Med. 2025 Sep 5;22(9):e1004728. doi: 10.1371/journal.pmed.1004728 (PMC12425212; doi:10.1371/journal.pmed.1004728)
Supplement: S7 Text — (DOCX) [file pmed.1004728.s009.docx]

**DRIFT-TRIAL PARTICIPATING STUDY CENTERS**

The DRIFT study was conducted in five trauma centres in the Nordic Countries. All participating centers are primary regional referral centers for orthopedic trauma patients in the area:

1. Tampere University Hospital

Elämänaukio 2, 33520, Tampere, Finland

2. Central Finland Central Hospital (current name Hospital Nova)

Hoitajantie 3, 40620 Jyväskylä, Finland

3. Satakunta Central Hospital

Sairaalantie 3, 28500 Pori, Finland

4. Karolinska University Hospital,

Hälsovägen 13, Huddinge, Sweden

5. Viborg Regional Hospital

Heibergs Alle 5A, 8800 Viborg, Denmark
