# Supplementary material for: Nonoperative treatment versus volar locking plating for distal radius fracture in patients aged 65 years or older (DRIFT trial): A randomized controlled trial
Source: PLoS Med. 2025 Sep 5;22(9):e1004728. doi: 10.1371/journal.pmed.1004728 (PMC12425212; doi:10.1371/journal.pmed.1004728)
Supplement: S8 Text — (PDF) [file pmed.1004728.s010.pdf]

DRIFT TRIAL - Conclusions

Version 1. A = operative, B = conservative, C = operative, D = conservative

In this NITEP collaboration randomized controlled trial, we found that operative treatment of primarily unstable distal radius fracture in patients aged 65 years or older may improve wrist function compared with non-operative treatment measured with PRWE in 12 months follow-up. The mean difference between the groups was, however, smaller than the predefined MCID of the PRWE (11points). This contrasts with previous studies that identified differences only at the first few months of the follow-up.

In patients with primarily acceptable fracture reduction and fracture malalignment in early follow up (5 to 10 days), operative treatment does not seem to provide improvement in wrist function measured with PRWE in 12 months follow-up. We found small difference in benefit for operative treatment, but since the 95% confidence intervals do not exclude the predefined MCID, we can not definitively rule out the benefit of operative treatment. Our results suggest that the decision of treatment modality should be made after the primary fracture reduction and after this there is no additional benefit from controlling the fracture position in terms of the expected wrist function.

Version 2. A = operative, B = conservative, C = conservative, D = operative

In this NITEP collaboration randomized controlled trial, we found that operative treatment of primarily unstable distal radius fracture in patients aged 65 years or older may improve wrist function compared with non-operative treatment measured with PRWE in 12 months follow-up. The mean difference between the groups was

, however, smaller than the predefined MCID of the PRWE (11points). This contrasts with previous studies that identified differences only at the first few months of the follow-up.

In patients with primarily acceptable fracture reduction and fracture malalignment in early follow up (5 to 10 days), operative treatment does not provide improvement in wrist function measured with PRWE in 12 months follow-up. We found small difference in benefit for non-operative treatment and the 95% confidence intervals exclude MCID in favor of operative treatment. Our results suggest that the decision of treatment modality should be made after the primary fracture reduction and after this there is no additional benefit from controlling the fracture position in terms of the expected wrist function.

Version 3. A = conservative, B = operative, C = operative, D = conservative

In this NITEP collaboration randomized controlled trial, we found that non-operative treatment of primarily unstable distal radius fracture in patients aged 65 years or older may improve wrist function compared with operative treatment measured with PRWE in 12 months follow-up. The mean difference between the groups was smaller than the predefined MCID of the PRWE (11points) but the 95% confidence intervals exclude MCID in favor of operative treatment. This contrasts with previous studies that have identified benefit for operative treatment in the first few months of the follow-up.

In patients with primarily acceptable fracture reduction and fracture malalignment in early follow up (5 to 10 days), operative treatment does not seem to provide improvement in wrist function measured with PRWE in 12 months follow-up. We found small difference in benefit for operative treatment, but since the 95% confidence intervals do not exclude the predefined MCID, we can not definitively rule out the benefit of operative treatment.Our results suggest that the decision of treatment modality should be made after the primary fracture reduction and after this there is no additional benefit from controlling the fracture position in terms of the expected wrist function.

Version 4. A = conservative, B = operative, C = conservative, D = operative

In this NITEP collaboration randomized controlled trial, we found that non-operative treatment of primarily unstable distal radius fracture in patients aged 65 years or older may improve wrist function compared with operative treatment measured with PRWE in 12 months follow-up. The mean difference between the groups was smaller than the predefined MCID of the PRWE (11points) but the 95% confidence exclude MCID in favor of operative treatment. This contrasts with previous studies that have identified benefit for operative treatment in the first few months of the follow-up.

In patients with primarily acceptable fracture reduction and fracture malalignment in early follow up (5 to 10 days), operative treatment does not seem to provide improvement in wrist function measured with PRWE in 12 months follow-up. We found small difference in benefit for non-operative treatment, and the 95% confidence intervals exclude MCID in favour of operative treatment. Our results suggest that the decision of treatment modality should be made after the primary fracture reduction and after this there is no additional benefit from controlling the fracture position in terms of the expected wrist function.

Tampere, Finland 30.11.2024

Professor Ville Mattila

Associate professor Antti Launonen

Associate professor Aleksi Reito

MD Teemu Hevonkorpi
