# Supplementary material for: Nonoperative treatment versus volar locking plating for distal radius fracture in patients aged 65 years or older (DRIFT trial): A randomized controlled trial
Source: PLoS Med. 2025 Sep 5;22(9):e1004728. doi: 10.1371/journal.pmed.1004728 (PMC12425212; doi:10.1371/journal.pmed.1004728)
Supplement: S9 Text — (DOC) [file pmed.1004728.s011.doc]

**NITEP *Nordic Radius*–study**

**Nordic Innovative Trial to Evaluate Osteoporotic Fractures: Non-operative Treatment Versus Surgery with Volar Locking Plate in the Treatment of Distal Radius Fracture in Patients Over Sixty Five Years– a Prospective, Randomized Controlled Trial**

**Participants of the Consortium**

**University of Tampere**

Project leader: Ville Mattila, MD, PhD, professor of orthopaedics and traumatology, University of Tampere School of Medicine and visiting professor at the Department of Clinical Science, Intervention and Technology (CLINTEC), Division of Orthopaedics and Biotechnology, Karolinska Institutet, Stockholm, Sweden

Address: Teiskontie 35, PL2000, 33521 Tampere, Finland

Tel: +358-3-311 67674

e-mail: [ville.mattila@uta.fi](mailto:ville.mattila@uta.fi)

Main Researcher: Antti Launonen, post-doc, Tampere University Hospital

Scientific advisor: Minna Laitinen, adjunct professor, Tampere University Hospital

Participating researchers (PhD students):

Bakir Sumrein

Teemu Hevonkorpi

Other participants: Heini Huhtala, statistical consultant, University of Tampere

**Jyväskylä Central Hospital**

Team leader: Toni Luokkala, MD, orthopaedics and traumatology, Jyväskylä Central Hospital, Jyväskylä, Finland

Participating researchers (Post-docs):

Aleksi Reito

**Satakunta Central Hospital**

Team Leader: Juha Kukkonen, MD, PhD, orthopaedics and traumatology, Satakunta Central Hospital, Pori, Finland

1. **BACKGROUND**

The impact of degenerative musculoskeletal diseases on individuals and on society as a whole is going to increase dramatically due to the increasing age and more sedentary lifestyle of western populations [1].

In the literature, it has been stated that a significant proportion of common medical interventions – including orthopaedics – are not based on solid high-quality scientific evidence, and additionally this evidence-based research has been stated to be “scandalously poor” [1,2]. Although there is a lack of supporting evidence, many interventions are still widely used. [3-5] There have been alarming reports showing that surgery on distal radius fractures (DRF) among others is increasing, even though there is no evidence to support the superiority of operative treatment over conservative treatment [3,4,6-9]. The surgical treatment of these orthopaedic procedures might not only be futile for individual patients, but they are quite often highly resource-consuming. As the economic situation has become more challenging in the Nordic countries, we are obliged to target our limited financial resources to treatments with proven efficacy and cost-effectiveness.

DRF is the most common upper extremity fracture [10]. The traditional way to classify DRFs has been to classify them as stable and unstable fractures. The treatment has followed this classification as a stable fracture maintains reduction during conservative treatment, whereas an unstable fracture will lose the reduction. Acceptable reduction is commonly achieved by closed reduction and non-operative treatment with cast immobilization. However, the reduction achieved might be lost during cast immobilization. This loss of reduction can occur early within the first two weeks or later at about 6 weeks.

The volar locking plate was introduced in the early 2000s. In theory, it provides a stable reconstruction that prevents the collapse of the dorsal cortex, and also allows early mobilization and rapid recovery as the wrist joint does not need immobilization [11]. Furthermore, it is thought that the reduction of the displaced fracture could decrease complications and yield a superior long-term functional outcome.

Several other surgical techniques such as metal wires, external fixators, dorsal plates, fragment specific plates and screws have been proposed as solutions for DRF fixation. Interestingly, none of the procedures has proved to be superior to the others in randomized controlled trial (RCT) settings [12-14]. According to high-quality RCTs, percutaneous techniques such as external fixator and metal wires produce a comparable functional outcome with the use of internal fixation with volar locking plate [12-14].

In the literature, there are numerous articles that compare different surgical procedures. However, the key question as to whether operative treatment and the better restoration of anatomy yields a better functional outcome when measured with patient reported outcome measures (PROMs) in the elderly population has remained unanswered. In addition to patient-related factors, the cost-effectiveness of the surgery is also unknown. Surgical treatment has been shown to result in a lower malunion rate than closed reduction and cast immobilization [15]. In addition, functional outcome of DRF has been shown to correlate with the anatomical restoration of the articular surface especially in young and middle-aged patients [16-18].

Several different radiographic parameters have been proposed as predictive factors for long-term functional outcome. Positive ulnar variance, dorsal angulation and step or gap in the articular surface affects the functional outcome among patients under 65 years [19-28]. To summarize, there is moderate evidence that in patients under 65 years the restoration of the anatomy is important [17,18]. However, based on several moderate-quality RCT’s, it seems that in elderly people (over 65 years) these anatomic parameters do not correlate with the functional outcome and that non-operative and operative treatment produces comparable results unrelated to the anatomy of the distal radius [29]. Moreover, non-operative treatment may be related to a lower rate of complications [30]. The weaknesses in these RCT’s were small sample size, heterogeneous primary outcome measures, short follow-up times and the inadequate reporting of complications. In fact, a recent Finnish trustworthy guideline ”Rannemurtuman Käypä hoito – suositus“ sums up the existing evidence and states that operative treatment of DRF’s in elderly patients (over 65 years), irrespective of fracture position, is not encouraged.

Even though there is no evidence to support the operative treatment of DRFs in elderly patients, many researchers have reported an increase in surgical activity [6,31,32]. Although the traditional treatment for DRFs has been non-operative with cast immobilization, a more than fivefold increase in the use of internal fixation has occurred since 1997 in the US [31]. There has also been a shift in Finland and Sweden during the last decade from non-operative treatment and external fixators to volar plating [6,7,32]. In fact, the incidence of the surgical treatment of DRF doubled between 1998 and 2008 [6,7,32].

In order to identify fractures with early instability in conservatively treated patients, various follow-up protocols have been introduced. The most commonly used follow-up protocol has included clinical visits at 1, 2 and 5 weeks with radiographs. However, the efficacy of these protocols and especially routinely taken radiographs can be challenged, as the existing literature suggests that re-intervention (closed re-reduction or conversion from conservative treatment to operative treatment) in cases of re-displacement do not produce better functional outcome in elderly patients (over 65 years) [25,33,34]. The rate of fractures healing in malposition during cast immobilization has been suggested to be as high as 30% to 60% of all DRFs [35-37].

As functional outcome correlates poorly with the anatomy restored after fracture healing, the high number of clinical visits with radiographs is a topic for further discussion. [19,30] Moreover, when the generality of DRF is taken into account, the number of these probably unnecessary follow-up visits constitutes a major economic burden.

Functional outcome and overall satisfaction in DRF patients can be affected by factors other than fracture or age. Pain catastrophizing and fear of using the injured limb are related to disability, increased pain and muscle weakness in upper extremity and DRF patients [38-40]. The severity of acute pain, catastrophic thinking and trauma related anxiety have been shown to be associated with finger stiffness after DRF [41]. The pain catastrophizing scale (PCS) is one of the most widely used measures of catastrophic thinking related to pain [42]. It has been translated into several languages and has been widely incorporated in the assessment protocol of pain clinics and rehabilitation centers. However, pain catastrophizing as a predictor of functional outcome has not previously been studied with elderly DRF patients treated non-operatively or operatively with volar locking plate.

1. **OBJECTIVES**

The present collaboration study on the treatment of distal radius fractures aims to achieve the following:

(i) To to compare non-operative treatment to volar plating in the treatment of initially malaligned distal radius fractures in patients aged 65 and older in terms of functional outcome measured with PRWE

(ii) To to compare non-operative treatment to volar plating in the treatment of distal radius fractures with early instability during follow-up, i.e., loss of reduction at 1 week (range 5 to 10 days) in patients aged 65 and older in terms of functional outcome measured with PRWE

(iii) To compare pain, disability, quality of life, grip strength and the number of complications after conservative treatment and initial and delayed operative treatment of distal radius fracture.

(iv) To assess the effect of pain catastrophizing score (PCS) on the functional outcome of non-operatively and operatively treated distal radius fracture

(v) To to assess the association between physical activity and the number of wrist movements measured with Axivity accelerometer and functional outcome measured with PROMs of non-operatively and operatively treated distal radius fractures

(vi) To assess the effect of initial as well as the final radiological parameters on the functional outcome

(vii) To assess the correlation of probability of radiological malalignment estimated by clinical prediction rule (EWC) with functional outcome measured with PRWE and PASS

1. **PRIMARY HYPOTHESES**

Our primary hypotheses are as follows:

(i) Initial operative treatment of distal radius fracture with volar locking plates does not yield superior results compared with non-operative treatment.

(ii) Late operative treatment of distal radius fracture with volar locking plates does not yield superior

results compared with continued non-operative treatment.

(iii) Initial or delayed operative treatment of malaligned distal radius fracture does not result in superior results compared with non-operative treatment with regard to pain, disability, quality of life, grip strength, and number of complications.

(iv) A high pain catastrophizing score predicts a poor functional outcome on the PRWE scale

(v) A high level of physical activity and a high number of wrist movements predict good functional

outcome on the PRWE scale and correlate negatively with the PCS.

(vi) Significant initial dislocation in radiographs (and high probability of malunion in EWC results) predicts a subset of poor functional outcomes on the PRWE scale and on PASS in patients between 65 to 74 years of age but not in patients aged 75 and older.

1. **STUDY DESIGN**

The study is a prospective, randomized, controlled, multi-center trial. The aim of the study is to compare the conservative and operative treatment of initially or early unstable distal radius fractures.

The primary outcome in this study is the PRWE score measured after one and two years [43]. The secondary outcomes measured are disability [Quick-DASH (disabilities of the arm, shoulder and arm)], pain in visual analogue scale (VAS), PCS, quality of life (15-D), physical activity and the number of wrist movements measured with Axivity accelerometer, complications and the number of surgical interventions in the conservatively managed group. Subgroup analysis will be performed to find out patient-specific features indicating good or worse outcome. PCS will be used to assess the presence of any mental susceptibility that may have a possible influence on the functional outcome [42]. The Axivity accelerometer will be used for the objective evaluation of the patient’s physical activity and movements of the fractured wrist in a subsample of patients. It will be used for four days at the 3-month and 1-year follow-up time points. Edinburgh Wrist Calculator (EWC) will be used to assess the probability of radiological malalignment from initial radiographs after injury, and its correlation with functional outcome and patient self-assessed state of symptoms. The degree of frailty among the patients in the study cohort will be assessed with Clinical Frailty Scale which is commonly used in geriatric medicine. Assessment of frailty is used to indicate if patients of certain frailty level benefit from operative or non-operative treatment more than patients of lower/higher frailty level.

1. **RESEARCH METHODS AND MATERIAL**
   1. **PATIENT SELECTION**

The eligible study population will consist of all consecutively treated patients (over 64 years) with a distal radius fracture identified in the public or referral emergency departments of participating hospitals.

The following criteria will be used throughout the study for patient selection:

Inclusion criteria:

- low energy intra or extra-articular dorsally displaced distal radius fracture within 3 cm of the radiocarpal joint, diagnosed with lateral and posterior-anterior radiographs in ER
- >10° dorsal tilt and/or over 2 mm step-off and/or over 3 mm shortening in the radiograph

Exclusion criteria:

- Refusal to participate in the intervention
- Open fracture more than Gustilo 1 gradus
- Age under 65 years
- Chauffeure’s or Barton´s fracture
- Smith´s fracture (volar angulation of the fracture)
- Does not understand written and spoken guidance in local languages
- Pathological fracture or previous fracture in the same wrist or forearm
  1. **RANDOMIZATION**

Patients will be randomized using a random number matrix in block allocation fashion. The blocks will be age, site and intra-extra articular-dependent because, based on the literature, age, presence of intra-articular fracture and functional outcome are associated. The treatment allocations from the matrix will be acquired from an online randomization system (http:// randomize.net), where the researcher logs in after written consent is obtained and receives the allocated intervention. The researchers will not have access to the allocation sequence. Physicians responsible for the interventions will not participate in collecting the primary outcomes during the follow-up. The research coordinator will monitor the study flow. An independent monitoring committee has been established.

- 1. **INTERVENTION**
     1. **Phase 1**

In general, patients visiting the ER with dislocated DRF will undergo a closed reduction under local anesthesia by means of a local infiltration of Lidocaine 1%. The technique of closed reduction will not be limited to some specific method. After reduction, radiographs will be taken to verify the position of the fracture.

Patients are asked to visit the outpatient clinic for treatment assessment 1 to 5 days after the reduction. During that visit, patients fulfilling the inclusion criteria will be asked to participate in the study and enrollment will be confirmed and informed consent obtained. The post-reduction radiograph will be analyzed during the visit, and patient allocation will be as follows; If satisfactory position is achieved, the patient will be allocated to Cohort 2 and conservative treatment will be performed. If satisfactory reduction is not achieved fulfilling the inclusion criteria, the patient will be allocated to Cohort 1. After allocation, the patient will be asked to fill in the PCS, 15-D, patient history, and self-assessment questionnaires.

In Cohort 1, the patient will be randomized to either conservative (=Arm 1) or operative treatment (=Arm 2). Patients allocated to conservative treatment will undergo a standard treatment protocol with a dorsal cast for 5 weeks. Patients allocated to operative treatment will undergo surgery with volar locking plate with modified Henry’s volar approach. A dorsal cast will be used for 2 weeks. After cast removal, a physiotherapist will guide the patients in carrying out non-weight bearing, full range of motion exercises that will be continued until the 5-week follow-up appointment. After this, progressive weight bearing active and passive exercises will be conducted. From week 5 in the non-operative arm, the exercises will follow a similar exercise protocol as in the operative group.

- - 1. **Phase 2**

After one week, patients allocated to Cohort 2 will visit the orthopedic outpatient clinic in the hospital where the treatment was initially started. If reduction is maintained, the patient will undergo standard follow-up visits (=Arm 4). If reduction is lost, to fulfill the inclusion criteria for surgery the patient will be asked to participate in phase 2 of this study. After the patient´s enrollment has been confirmed and informed, written consent obtained, the patient will be randomized to either conservative (=Arm 3N) or operative (=Arm 3O) treatment. If allocated to conservative treatment, the patient will undergo the same protocol as those patients in Arm 1. Patients allocated to operative treatment will undergo surgery with volar locking plate with standard volar approach. In addition, physiotherapy and exercises will be conducted as described above.

Patients declining to attend the intervention trial in treatment assessment visit will be asked to join the external follow-up group. This group will be used as external validation; the group content and outcomes will be compared with the allocated intervention and control groups. The treatment will be carried out in line with normal clinical practice, but the patients will have the same follow-up and asked to fill in the same questionnaires as the allocated patients.

- 1. **FOLLOW-UP**

Conservatively treated patients in Arm 1 will be treated with cast immobilization. After 5 weeks, the cast will be removed and the patient will visit an orthopaedic outpatient clinic at the hospital for a follow-up visit. During the visit, direct lateral and anteroposterior radiographs will be taken and. The patient will again visit the outpatient clinic after three months and follow-up assessment will be performed by completing the PRWE, QuickDASH, VAS, PCS, and 15-D questionnaires. Radiographs will also be taken. Patient’s physical activity and the number of movements of the injured wrist will be evaluated with Axivity accelerometer in a subsample of patients (n=80). Operatively treated patients will visit the clinic at 5 weeks and at 3 months and the same follow-up protocol will be performed.

After the first week’s visit to the clinic for phase 2 patients, the same follow-up protocol as used in Cohort 1 will be performed.

Rehabilitation will be carried out after standardized, written protocol.


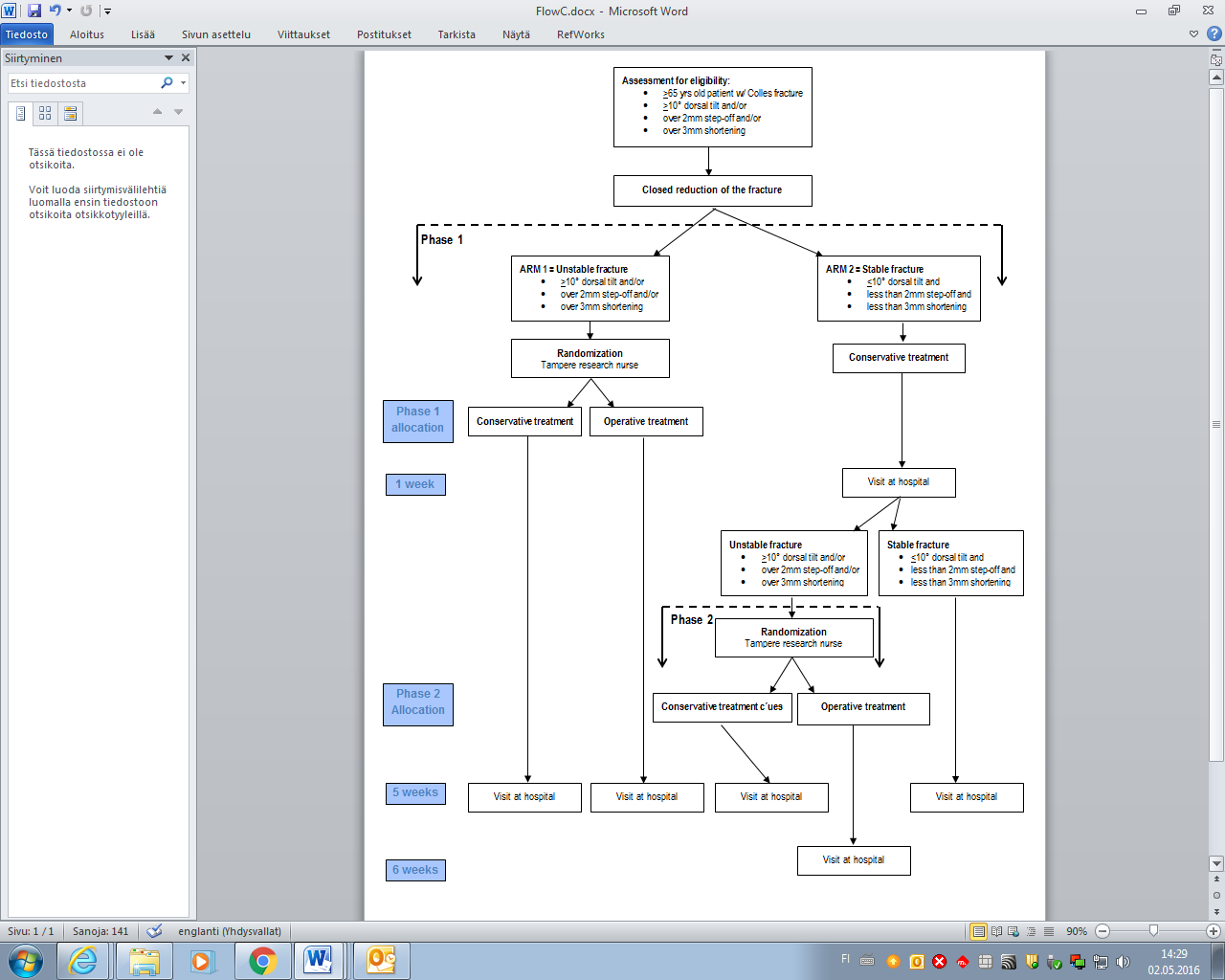


For all patients, PRWE is the primary outcome and will be measured at the 1 and 2-year follow-up time points by the Internet, post or telephone query. PCS, pain, Quick-DASH,15-D, and self-assessment will be measured (Table 1). At the 1-year follow-up, physical activity and the number of wrist movements will be measured with Axivity accelerometer in a subgroup of patients. Grip strength will also be measured.

At the 1 and 2-year follow-up, complications will be recorded by patient records.

**Table 1. Assessments and procedures**

|  | **Radiograph** | **PRWE** | **PCS** | **Pain** | **Grip** | **Axivity** | **Quick-DASH** | **15-D** | ***Clinical frailty scale*** | **Self-assessment** |
| --- | --- | --- | --- | --- | --- | --- | --- | --- | --- | --- |
| **Baseline** | **x** |  | **x** |  |  |  |  | **x** |  | **x** |
| **1 week (Cohort 2)** | **x** |  |  |  |  |  |  |  |  |  |
| **5-6 weeks** | **x** |  |  |  |  |  |  |  |  |  |
| **3 months** | **x** | **x** | **x** | **x** | **x** | **x** | **x** | **x** | **x** |  |
| **1 year** | **x** | **x** | **x** | **x** | **x** | **x** | **x** | **x** |  | **x** |
| **2 years** |  | **x** | **x** | **x** |  |  | **x** | **x** |  | **x** |

- 1. **POWER ANALYSIS**

In this trial, a validated wrist specific PRWE-score will be used as the main outcome measure. Recently, Wallenkamp and coworkers reported that the minimal clinically important difference in PRWE is 11 points and SD is 14 [43]. Based on power calculations (Cl 95%, power 0.95, SD 14), the required sample size per group is 40 patients. Assuming 30% drop-out based on possible surgical intervention during cast treatment, group size would be 57 per group (total 114). Taking five arms into account, a total of 285 patients is needed. Should patients change to a different treatment group (cross-overs), they will still be analyzed according to the intention-to-treat principle.

- 1. **DATA MANAGEMENT PLAN**

Each patient will be assigned a unique trial identification number (TIN) that will be matched with the patient’s identification number (ID). The matching key will be stored in a locked office at Tampere University Hospital, Finland, and the identification of each patient will only be possible after retrieving the matching key. Throughout the trial, the research data will be handled only with a TIN. The research data will be saved to a database with an online patient management program (PMP) located on a secure research server. The research data saved to the server will contain only anonymous TINs with a set of numbers acquired from the questionnaires, i.e., each question will be answered with a number. This will ensure the anonymity of each individual patient and that the identity of the patient will remain secret should the server data be revealed to third-parties.

All primary and secondary data will be acquired and stored on the study trial server. Data will be entered either by the patient during the control visits (via tablets) or by a researcher or study nurse when the questionnaires are mailed. The researchers from each hospital will have access to the secure study server where the trial research data is stored. The server has been approved by an information security committee at Tampere University Hospital. At the end of the trial, each researcher will have access to the data for further analyses.  The questionnaires will be pre-programmed according to the PMP, and the individual patient data acquired at different time-points will be saved in Comma Separated Values (CSV) format, which is transferrable to, e.g., Microsoft Excel. All variables in the dataset will be described and suitable metadata standards will be used when available.

The copyright of the trial research data will be owned and created by the collaboration parties. The data will be shared freely among the collaboration parties. All participating researchers will receive access to the data after the trial is completed. Due to confidentiality and legal agreements, public data sharing will be restricted, (we have permission to store the data in the specific research server, but not to transfer the data). Under certain circumstances, e.g., when a new member joins the collaboration, we will grant access to the data. All data will be saved for five years after the end of the trial.

- 1. **STATISTICAL ANALYSIS**

Differences between groups in continuous skewed main outcome variables will be analyzed by the Mann-Whitney U-test, and analyzed by the t-test when the variables are unskewed. The results will be presented with 95% confidence intervals. Two-way-tables with the chi-square test will be used for dichotomous variables. In subgroup analysis, the effect of age, sex, fracture group, smoking and other diseases will be evaluated against the scores and overall quality of life after fracture. A p-value of less than 0.05 is considered significant.

Analysis of covariance will be used to assess the effect of pain catastrophizing score on the outcome of cast treatment. PRWE will be used as a dependent variable, cast treatment as independent and pain catastrophizing score as covariate.

The effect of cast immobilization treatment on the PRWE will also be investigated in the multivariate manner. Multivariable analysis will be performed with linear regression analysis since the outcome variable PRWE is normally distributed. The main variable of interest included will be cast immobilization. Age, sex, fracture group, smoking and other diseases will be used as confounding variables.

- 1. **Patient involvement in trial**

In order to improve patient involvement in the trial, we will interview patients with distal radius fracture before the inset of the trial. The aim of the interviews will be to move towards patient-centered medicine by taking into account the goals, preferences and values of patients. We will involve patients by asking questions in the beginning of the treatment in order to identify the questions to ask and the outcomes to measure. The interviews will be repeated after one year, and the difference or indifference between the primary and follow-up responses will be reported.

- 1. **ETHICAL ISSUES**

Ethical approval for this study has been granted by the Regional Ethical Committee of Tampere University Hospital (ETL 16105).

1. **IMPLEMENTATION**
   1. **CONTINGENCY PLAN**

For the study, we will need to recruit more than 200 patients. Recruitment for arm 2, phase 2, will be challenging. As a single center, there is always the risk that recruiting the required number of patients will not be achieved. This risk will be averted through Nordic collaboration by means of a multicenter study. We have homogeneity between centers with solid trial designs, data and project management including monitoring. Site personnel training has been carried out. We have been assisted by local research centers. The stability of the trial has been maintained with regular communication where researchers have shared concerns, problems and successes. Regular biannual meetings (next on Aug 24, 2017 in Tampere) will motivate the research team and maintain stable quality in treatment. The meetings also include presentations of future studies and trials, methodology and scientific meanings will be debated. The homepage of the current trial has been launched (www.nitep.eu).

- 1. **JUSTIFICATION FOR THE PROPOSED RESEARCH INFRASTRUCTURE**

As shown earlier, a significant proportion of common orthopaedic interventions are not based on high-quality scientific evidence [2,44]. To date, Nordic countries have not been seen as entities with similar practices in operative treatment and high quality registers. Thus, no cross-border collaboration between Nordic countries similar to the one proposed in this application has existed before.

Previously, our NITEP group has shown that such Nordic collaboration can work. The project leader, professor Ville Mattila from Tampere University and Tampere University Hospital, has been investigating treatment policies for common musculoskeletal injuries since 2008 using data from the Finnish National Hospital Register. More recently, he has integrated Swedish trends in collaboration with professor Li Felländer-Tsai (Karolinska Institutet, Stockholm) during his post-doc period at the Karolinska Hospital during 2013 and 2014 [3,6,45-49]. MD, PhD Antti Launonen’s PhD project on proximal humerus fractures made it possible to create a unique Nordic collaboration network with which to conduct these ambitious aims. Docent Hans Berg, MD Kennet Jansson, docent Inger Mechelunburg and PhD Kristo Kask made it possible to conduct this project. In fact, to date, 20^th^ April 2017 2NN?/298 patients have been recruited to our ongoing proximal humerus fracture study. Our proximal humerus study was granted funding by the Finnish Academy in 2013 (Laitinen, 2013).

1. **BUDGET**

This trial has received a research grant from the Academy of Finland. Other Nordic collaborators are responsible for their own costs.

1. **Expected results and impact**
2. We expect to observe similar functional outcomes after conservative and operative treatment in displaced distal radius fracture.
3. We expect that functional outcome after treatment of distal radius fracture is related more to catastrophic thinking than with treatment modality.
4. We assume that the late operative treatment of displaced distal radius fractures observed during short-term follow up will have no additional benefit to continued conservative treatment.
5. After publishing these results, we aim to support the creation and dissemination of trustworthy guidelines by health authorities and professional organizations.
6. We expect that after demonstrating clinical results (our RCT’s) and supporting the creation and dissemination of trustworthy guidelines for treatment policies of these common orthopaedic complaints, Nordic countries will change substantially and reflect best current evidence.
7. This initiative will certainly result in the creation of an independent, scientist-driven Nordic research infrastructure capable of evaluating treatments for common musculoskeletal disorders

References

1. Woolf AD, Pfleger B. Burden of major musculoskeletal conditions. Bull World Health Organ. 2003;81: 646-656.

2. Lohmander LS, Roos EM. The evidence base for orthopaedics and sports medicine. BMJ. 2015;350: g7835.

3. Huttunen TT, Launonen AP, Pihlajamaki H, Kannus P, Mattila VM. Trends in the surgical treatment of proximal humeral fractures - a nationwide 23-year study in Finland. BMC Musculoskelet Disord. 2012;13: 261-2474-13-261.

4. Launonen AP, Lepola V, Flinkkila T, Laitinen M, Paavola M, Malmivaara A. Treatment of proximal humerus fractures in the elderly: a systemic review of 409 patients. Acta Orthop. 2015;86: 280-285.

5. Jarvinen TL, Sihvonen R, Englund M. Arthroscopy for degenerative knee--a difficult habit to break? Acta Orthop. 2014;85: 215-217.

6. Mattila VM, Huttunen TT, Sillanpaa P, Niemi S, Pihlajamaki H, Kannus P. Significant change in the surgical treatment of distal radius fractures: a nationwide study between 1998 and 2008 in Finland. J Trauma. 2011;71: 939-42; discussion 942-3.

7. Mellstrand-Navarro C, Pettersson HJ, Tornqvist H, Ponzer S. The operative treatment of fractures of the distal radius is increasing: results from a nationwide Swedish study. Bone Joint J. 2014;96-B: 963-969.

8. Fjalestad T, Hole MO, Hovden IA, Blucher J, Stromsoe K. Surgical treatment with an angular stable plate for complex displaced proximal humeral fractures in elderly patients: a randomized controlled trial. J Orthop Trauma. 2012;26: 98-106.

9. Olerud P, Ahrengart L, Ponzer S, Saving J, Tidermark J. Internal fixation versus nonoperative treatment of displaced 3-part proximal humeral fractures in elderly patients: a randomized controlled trial. J Shoulder Elbow Surg. 2011;20: 747-755.

10. Larsen CF, Lauritsen J. Epidemiology of acute wrist trauma. Int J Epidemiol. 1993;22: 911-916.

11. Beharrie AW, Beredjiklian PK, Bozentka DJ. Functional outcomes after open reduction and internal fixation for treatment of displaced distal radius fractures in patients over 60 years of age. J Orthop Trauma. 2004;18: 680-686.

12. Esposito J, Schemitsch EH, Saccone M, Sternheim A, Kuzyk PR. External fixation versus open reduction with plate fixation for distal radius fractures: a meta-analysis of randomised controlled trials. Injury. 2013;44: 409-416.

13. Walenkamp MM, Bentohami A, Beerekamp MS, Peters RW, van der Heiden R, Goslings JC, et al. Functional outcome in patients with unstable distal radius fractures, volar locking plate versus external fixation: a meta-analysis. Strategies Trauma Limb Reconstr. 2013;8: 67-75.

14. Costa ML, Achten J, Parsons NR, Rangan A, Griffin D, Tubeuf S, et al. Percutaneous fixation with Kirschner wires versus volar locking plate fixation in adults with dorsally displaced fracture of distal radius: randomised controlled trial. BMJ. 2014;349: g4807.

15. MacDermid JC, Roth JH, Richards RS. Pain and disability reported in the year following a distal radius fracture: a cohort study. BMC Musculoskelet Disord. 2003;4: 24.

16. Wilcke MK, Abbaszadegan H, Adolphson PY. Patient-perceived outcome after displaced distal radius fractures. A comparison between radiological parameters, objective physical variables, and the DASH score. J Hand Ther. 2007;20: 290-8; quiz 299.

17. Merchan EC, Breton AF, Galindo E, Peinado JF, Beltran J. Plaster cast versus Clyburn external fixation for fractures of the distal radius in patients under 45 years of age. Orthop Rev. 1992;21: 1203-1209.

18. Rodriguez-Merchan EC. Plaster cast versus percutaneous pin fixation for comminuted fractures of the distal radius in patients between 46 and 65 years of age. J Orthop Trauma. 1997;11: 212-217.

19. Grewal R, MacDermid JC. The risk of adverse outcomes in extra-articular distal radius fractures is increased with malalignment in patients of all ages but mitigated in older patients. J Hand Surg Am. 2007;32: 962-970.

20. Leung F, Ozkan M, Chow SP. Conservative treatment of intra-articular fractures of the distal radius--factors affecting functional outcome. Hand Surg. 2000;5: 145-153.

21. Trumble TE, Schmitt SR, Vedder NB. Factors affecting functional outcome of displaced intra-articular distal radius fractures. J Hand Surg Am. 1994;19: 325-340.

22. Batra S, Gupta A. The effect of fracture-related factors on the functional outcome at 1 year in distal radius fractures. Injury. 2002;33: 499-502.

23. Warwick D, Field J, Prothero D, Gibson A, Bannister GC. Function ten years after Colles' fracture. Clin Orthop Relat Res. 1993;(295): 270-274.

24. Villar RN, Marsh D, Rushton N, Greatorex RA. Three years after Colles' fracture. A prospective review. J Bone Joint Surg Br. 1987;69: 635-638.

25. McQueen MM, Hajducka C, Court-Brown CM. Redisplaced unstable fractures of the distal radius: a prospective randomised comparison of four methods of treatment. J Bone Joint Surg Br. 1996;78: 404-409.

26. Karnezis IA, Panagiotopoulos E, Tyllianakis M, Megas P, Lambiris E. Correlation between radiological parameters and patient-rated wrist dysfunction following fractures of the distal radius. Injury. 2005;36: 1435-1439.

27. Knirk JL, Jupiter JB. Intra-articular fractures of the distal end of the radius in young adults. J Bone Joint Surg Am. 1986;68: 647-659.

28. Kelly AJ, Warwick D, Crichlow TP, Bannister GC. Is manipulation of moderately displaced Colles' fracture worthwhile? A prospective randomized trial. Injury. 1997;28: 283-287.

29. Nelson GN, Stepan JG, Osei DA, Calfee RP. The impact of patient activity level on wrist disability after distal radius malunion in older adults. J Orthop Trauma. 2015;29: 195-200.

30. Arora R, Lutz M, Deml C, Krappinger D, Haug L, Gabl M. A prospective randomized trial comparing nonoperative treatment with volar locking plate fixation for displaced and unstable distal radial fractures in patients sixty-five years of age and older. J Bone Joint Surg Am. 2011;93: 2146-2153.

31. Chung KC, Shauver MJ, Birkmeyer JD. Trends in the United States in the treatment of distal radial fractures in the elderly. J Bone Joint Surg Am. 2009;91: 1868-1873.

32. Wilcke MK, Hammarberg H, Adolphson PY. Epidemiology and changed surgical treatment methods for fractures of the distal radius: a registry analysis of 42,583 patients in Stockholm County, Sweden, 2004-2010. Acta Orthop. 2013;84: 292-296.

33. Roumen RM, Hesp WL, Bruggink ED. Unstable Colles' fractures in elderly patients. A randomised trial of external fixation for redisplacement. J Bone Joint Surg Br. 1991;73: 307-311.

34. McQueen MM, MacLaren A, Chalmers J. The value of remanipulating Colles' fractures. J Bone Joint Surg Br. 1986;68: 232-233.

35. Leone J, Bhandari M, Adili A, McKenzie S, Moro JK, Dunlop RB. Predictors of early and late instability following conservative treatment of extra-articular distal radius fractures. Arch Orthop Trauma Surg. 2004;124: 38-41.

36. Wadsten MA, Sayed-Noor AS, Englund E, Buttazzoni GG, Sjoden GO. Cortical comminution in distal radial fractures can predict the radiological outcome: a cohort multicentre study. Bone Joint J. 2014;96-B: 978-983.

37. Altissimi M, Mancini GB, Azzara A, Ciaffoloni E. Early and late displacement of fractures of the distal radius. The prediction of instability. Int Orthop. 1994;18: 61-65.

38. Das De S, Vranceanu AM, Ring DC. Contribution of kinesophobia and catastrophic thinking to upper-extremity-specific disability. J Bone Joint Surg Am. 2013;95: 76-81.

39. Linton SJ, Nicholas MK, MacDonald S, Boersma K, Bergbom S, Maher C, et al. The role of depression and catastrophizing in musculoskeletal pain. Eur J Pain. 2011;15: 416-422.

40. Jelicic M, Kempen GI. Do psychological factors influence pain following a fracture of the extremities? Injury. 1999;30: 323-325.

41. Teunis T, Bot AG, Thornton ER, Ring D. Catastrophic Thinking Is Associated With Finger Stiffness After Distal Radius Fracture Surgery. J Orthop Trauma. 2015;29: e414-20.

42. Sullivan MJL, Bishop SR, Pivik J. The Pain Catastrophizing Scale: Development and validation. Psychol Assess. 1995;7: 524-532.

43. Walenkamp MM, de Muinck Keizer RJ, Goslings JC, Vos LM, Rosenwasser MP, Schep NW. The Minimum Clinically Important Difference of the Patient-rated Wrist Evaluation Score for Patients With Distal Radius Fractures. Clin Orthop Relat Res. 2015;473: 3235-3241.

44. Lim HC, Adie S, Naylor JM, Harris IA. Randomised trial support for orthopaedic surgical procedures. PLoS One. 2014;9: e96745.

45. Huttunen TT, Kannus P, Lepola V, Pihlajamaki H, Mattila VM. Surgical treatment of clavicular fractures in Finland - A register based study between 1987 and 2010. Injury. 2013;44: 1899-1903.

46. Huttunen TT, Kannus P, Lepola V, Pihlajamaki H, Mattila VM. Surgical treatment of humeral-shaft fractures: a register-based study in Finland between 1987 and 2009. Injury. 2012;43: 1704-1708.

47. Mattila VM, Huttunen TT, Haapasalo H, Sillanpaa P, Malmivaara A, Pihlajamaki H. Declining incidence of surgery for Achilles tendon rupture follows publication of major RCTs: evidence-influenced change evident using the Finnish registry study. Br J Sports Med. 2015;49: 1084-1086.

48. Huttunen TT, Kannus P, Rolf C, Fellander-Tsai L, Mattila VM. Acute achilles tendon ruptures: incidence of injury and surgery in Sweden between 2001 and 2012. Am J Sports Med. 2014;42: 2419-2423.

49. Nordenvall R, Bahmanyar S, Adami J, Mattila VM, Fellander-Tsai L. Cruciate ligament reconstruction and risk of knee osteoarthritis: the association between cruciate ligament injury and post-traumatic osteoarthritis. a population based nationwide study in Sweden, 1987-2009. PLoS One. 2014;9: e104681.
