# Supplementary material for: Nonoperative treatment versus volar locking plating for distal radius fracture in patients aged 65 years or older (DRIFT trial): A randomized controlled trial
Source: PLoS Med. 2025 Sep 5;22(9):e1004728. doi: 10.1371/journal.pmed.1004728 (PMC12425212; doi:10.1371/journal.pmed.1004728)
Supplement: S10 Text — (DOCX) [file pmed.1004728.s012.docx]

**DRIFT TRIAL - Metadata**

**Primary and secondary outcomes of the DRIFT trial**

| **Outcome** |  |
| --- | --- |
| Patient rated wrist evaluation (PRWE) | The PRWE is a 15-item questionnaire designed to measure wrist pain and disability in activities of daily living.  PRWE allows patients to rate their levels of wrist pain and disability from 0 to 10, and consists of 2 subscales:  Pain subscale: contains 5 items each of which is further rated from 1-10. The maximum score in this section is 50 and minimum 0  Function subscale: contains total 10 items which are further divided into 2 sections i.e specific activities (having 6 items) and usual activities (having 4 items). The maximum score in this section is 50 and minimum 0.  Time points: 3 months, 12 months, 24 months |
| Quick Disabilities of Arm, Shoulder and Hand (QuickDASH) | The QuickDASH is a shortened version of the DASH Outcome Measure. Instead of 30 items, the QuickDASH uses 11 items to measure physical function and symptoms in people with any or multiple musculoskeletal disorders of the upper limb. The QuickDASH is scored in two components: the disability/symptom section (11 items, scored 1-5) and the optional high performance sport/music or work modules (four items, scored 1-5).  Time points: 3 months, 12 months, 24 months |
| Fifteen dimensional (15-D) | The 15D is a generic, comprehensive (15-dimensional), self-administered instrument for measuring health-related quality of life among adults (age 16+ years). It combines the advantages of a profile and a preference-based, single index measure. A set of utility or preference weights is used to generate the 15D score (single index number) on a 0-1 scale.  Time points: 3 months, 12 months, 24 months |
| Visual Analogue Scale (VAS) | The pain VAS is a unidimensional measure of pain intensity, used to record patients’ pain progression, or compare pain severity between patients with similar conditions. The most simple VAS is a straight horizontal line of fixed length, usually 100 mm. The ends are defined as the extreme limits of the parameter to be measured (pain) orientated from the left (worst) to the right (best).  Time points: 3 months, 12 months, 24 months |
| Pain catastrophizing scale (PCS) | The pain catastrophizing scale (PCS) is one of the most widely used measures of catastrophic thinking related to pain. I The PCS consists of 13 statements containing a number of thoughts and feelings one may experience when having pain. The items are divided into the categories of rumination, magnification and helplessness, with each item scored on a 5-point scale. The overall score has a range of 0-52  Time points: 3 months, 12 months, 24 months |
| Grip strength | Grip strenght was measured with Jamar Hydraulic Hand Dynamometer and compared to the healthy upper limb. The reported value was the difference between injured and non-injured upper limb (delta grip).  Time points: 3 months, 12 months |
| Clinical Frailty Scale | The Clinical Frailty Scale (CFS) is a straightforward and accessible tool that can be used to quickly and simply assess patients living with frailty. It has been validated in adults aged over 65 years. A score from 1 (very fit) to 9 (terminally ill) is given based on the descriptions and pictographs of activity and functional status.  Time points: 3 months |
| Self-assessment questionnaire | To improve patient involvement in this trial, we interviewed patients with DRF before the onset of the trial. The aim of the interviews was to move towards more patient-centered medicine by taking into account the patients’ preferences and beliefs for a good outcome. We asked the same questions at the beginning of the treatment and at the 12-month follow-up.  Time points: baseline, 12 months, 24 months |
| Axivity accelerometer | The Axivity accelerometer is a movement sensor measuring linear acceleration and angular velocity at high precision. Axivity was used for the objective evaluation of the patient’s physical activity and movements of the fractured wrist in a subsample of patients. It was used for four days at the 3-month and 1-year follow-up time points.  Time points: 3 months, 12 months |
| Dorsal angulation of the fracture | Dorsal angulation angle of the distal radius, reported in degrees.  Time points: baseline, 1 week (Cohort 2), 3 months, 12 months |
| Step/gap of the fracture | Step or gap on the articular surface of distal radius in the radiocarpal joint, reported in millimeters. Time points: baseline, 1 week (Cohort 2), 3 months, 12 months |
| Intra/extra articularity of the fracture | Presence of intra-articular fracture component in radiographs. Time points: baseline, 1 week (Cohort 2), 3 months, 12 months |
| Shortening of the radius | Shortening of the distal radius in comparison to articular surface of the ulna in ulnocarpal joint. Neutral ulnar variance was assumed. Results reported in millimeters. Time points: baseline, 1 week (Cohort 2), 3 months, 12 months |
| Adverse event | Any adverse event requiring treatment in the hospital during the follow-up period. Classified as adverse events (AE) and serious adverse events (SAE) and treatment-related or other adverse events. Time points: 12 months, 24 months |
