# Supplementary material for: Nonoperative treatment versus volar locking plating for distal radius fracture in patients aged 65 years or older (DRIFT trial): A randomized controlled trial
Source: PLoS Med. 2025 Sep 5;22(9):e1004728. doi: 10.1371/journal.pmed.1004728 (PMC12425212; doi:10.1371/journal.pmed.1004728)
Supplement: S11 Text — (DOCX) [file pmed.1004728.s013.docx]

**DRIFT TRIAL – Detailed information of volar locking plates used**

**Details of volar locking plates used in study centers**

| **Study center** | Distal radius VLP system | Number of patients |
| --- | --- | --- |
| Tampere University Hospital | Acu-Loc 2 Wrist Plating System  (Acumed, Hillsboro, OR, USA)  Ixos Radius Plating System  (KLS Martin, Tuttlingen, Germany) | 31/96 (32%)  8/96 (8%) |
| Viborg Regional Hospital | Acu-Loc 2 Wrist Plating System  (Acumed, Hillsboro, OR, USA) | 16/96 (17%) |
| Central Finland Central Hospital | Acu-Loc 2 Wrist Plating System  (Acumed, Hillsboro, OR, USA)  Medartis Aptus Wrist Distal Radius System  (Medartis, Basel, Switzerland) | 12/96 (13%)  8/96 (8%) |
| Satakunta Central Hospital | Acu-Loc 2 Wrist Plating System  (Acumed, Hillsboro, OR, USA) | 10/96 (10%) |
| Karolinska Institute Huddinge | Acu-Loc 2 Wrist Plating System  (Acumed, Hillsboro, OR, USA) | 11/96 (11%) |
