# Supplementary material for: Nonoperative treatment versus volar locking plating for distal radius fracture in patients aged 65 years or older (DRIFT trial): A randomized controlled trial
Source: PLoS Med. 2025 Sep 5;22(9):e1004728. doi: 10.1371/journal.pmed.1004728 (PMC12425212; doi:10.1371/journal.pmed.1004728)
Supplement: Attachment — Submitted filename: DRIFT-trial_Response to reviewers_final.docx [file pmed.1004728.s016.docx]

**Point-by-point responses**

Dear Editor:

We would like to take this opportunity to thank the Editorial team and the Reviewers for their constructive criticism of our manuscript. We have strived to revise the manuscript in accordance with the valuable comments received. As a result, we feel that the quality of our manuscript has vastly improved.

Please find below a specific list of the changes that we have made based on the recommendations we have received.

-----------------------------------------------------------

Comments from the editorial team:

We believe that the manuscript is potentially too complex for a nonspecialist audience. When revising it, please keep in mind that it should be accessible to a broad medical audience.

***Response:*** *Thank you for this important notice. When revising the manuscript, we kept in mind that the manuscript should be easily understandable to all readers of the PLOS Medicine.*

-----------------------------------------------------------
Comments from the reviewers:

Reviewer #1:

Clarity and Language

Simplify complex or repetitive sentences (e.g., in abstract and discussion).

***Response:*** *Thank you for the comment. When revising the manuscript, we aimed to amend the text so that there would not be unnecessary repetition or too complex sentences.*

Improve flow and readability, especially in the introduction.

***Response:*** *Thank you for the comment. When revising the manuscript, we aimed to improve the flow and readability of the text, especially in the introduction section.*

Limitations and Statistical Interpretation
Explicitly discuss the risk of type II error in the early malalignment subgroup.

***Response:*** *In the early malaligned group, sample size was smaller than in the primarily malaligned group. There is always a possibility for type II error. However, our sample size was enough to detect a standardized mean difference of 0.6 which is reasonable low and deemed relevant. Naturally larger sample size would have provided narrower confidence intervals.*

Consider including a post hoc power analysis or Bayesian interpretation for underpowered comparisons.

***Response:*** *We thank for the comment. Firstly, post hoc power analysis is not feasible or informative as post-hoc power is in 1:1 relation to p-value. This statistical fact has been documented numerous times in the professional literature. Please see eg. a classic article from 2001 where authors conclude “This approach, which appears in various forms, is fundamentally flawed.” (*[*https://www.tandfonline.com/doi/abs/10.1198/000313001300339897*](https://www.tandfonline.com/doi/abs/10.1198/000313001300339897)*)
Secondly, Bayesian interpretation would certainly be an excellent approach for a statistical approach. This paradigm gives very pragmatic results, such as the probability of achieving MCID, which are not possible in the frequentist paradigm. Our statistical analysis plan was based purely on a frequentist paradigm, and changing the whole approach at this point does not seem feasible. But a later secondary analysis using a Bayesian approach would be an interesting topic!*

Generalizability
Acknowledge that the Nordic setting may limit applicability in less-resourced healthcare environments.

***Response:*** *Thank you for this important notice. Indeed, there are major disparities in healthcare resources globally, and it may be difficult to apply the results in environments with less resources allocated to the treatment of upper-limb traumas. This important point has now been added to the Discussion, limitations section: “In addition, the Nordic setting of the trial may limit applicability of the results in less-resourced healthcare environments.”*

Adverse Events Reporting
Clarify which adverse events are treatment-related vs. unrelated.

***Response:*** *Thank you for the comment. The adverse events have now been classified as treatment-related adverse events or adverse events unrelated to received treatment in Table 3 of the manuscript.*

Consider using a standard grading system (e.g., Clavien-Dindo) for complications.

***Response:*** *Thank you for the comment. The Clavien-Dindo classification for postoperative complications has now been added to Supporting information S4 DRIFT-trial_adverse events.*

Figures and Tables
Improve font size and clarity in the flowchart (Fig 1).

***Response:*** *Thank you for the comment. The font size and clarity of the flowchart have now been improved.*

Use colorblind-friendly design and clearer axis labels in Fig 2 (e.g., note that lower PRWE = better function).

***Response:*** *Thank you for the important notice. The colors of Fig 2. have now been amended to colorblind-friendly design, and a following mention has been added to the figure legend: “PRWE is scaled from 0 to 100 with the minimum (=best) score of 0.”*

Ensure consistent naming of trial arms across text and visuals.

***Response:*** *Thank you for this comment. The naming of trial arms has now been checked for consistency.*

Reproducibility
Provide more surgical detail (plate type, reduction criteria) in the supplementary materials.

***Response:*** *Thank you for the comment. The details of used plates have been added to supporting information, S13 DRIFT-trial details of volar locking plates.*

Interpretation and Conclusions
Emphasize the marginal clinical significance of VLP benefits in primarily malaligned fractures.

***Response:*** *Thank you for the comment. The conclusion has now been amended as follows: “Operative treatment of primarily malaligned DRF with VLP may* ***slightly*** *improve wrist function at 12 months”*

Highlight potential clinical implications: reduced need for early radiographic follow-up in some cases.

***Response:*** *Thank you for the comment. The following sentence has now been added to the Abstract, Conclusions and the last chapter of Discussion section: “This questions the need for early radiographic follow-up during the non-operative treatment.”*

Abstract and Title
Clarify in the abstract that observed benefits were below the MCID.

***Response:*** *Thank you for the comment. The mention of mean difference being below the MCID of the PRWE in primarily malaligned DRFs has been now added to Methods and Findings section of the abstract, and has been kept unamended in Conclusions section of the abstract: “In primarily malaligned DRFs, the estimated mean effect for PRWE at 12 months was -9.6 points (95% CI [-17.4, -1.7]) in favor of VLP,* ***which is smaller than the predefined MCID of the PRWE (11 points).”***

Consider revising the title to include "randomized controlled trial" for clarity.

***Response:*** Thank you for the comment. The title has now been revised as follows: “DRIFT trial: Nonoperative treatment vs volar locking plating for distal radius fracture in patients aged 65 years or older: A randomized controlled trial”

Consistency
Standardize age group descriptions (e.g., use consistent phrasing for ">75" vs "older than 75").

***Response:*** *Thank you for the remark. The age group descriptions have now been standardized as “aged from 65 to 74 years” and “aged 75 years or older”and checked for consistency.*

Reviewer #2:
This manuscript reports the findings of the DRIFT trial. It is a well conducted trial and the reporting is also good. There are minor points made below but there are no major flaws in the study.

***Response:*** *Thank you for the positive feedback.*

1.      Introduction. The comments "existing RCT level evidence does not address the management of those DRFs that lose alignment during nonoperative treatment" and "To our knowledge, no previous RCTs have compared operative and nonoperative treatment in cases of early loss of alignment" are not correct. For example, the trial by Arora et al only included people with "inadequate initial reduction" and "loss of reduction at one week" i.e., patients who "lose alignment during non-operative treatment", which directly contradicts the statements in the Introduction. In fact, the inclusion criteria in the Arora study look the same as the current study.

***Response:*** *Thank you for the comment. It is true that there have been previous randomized controlled trials that have also had patients with early loss of fracture alignment. However, these studies have also had patients with primarily malaligned fractures, and the patients have been analyzed together / in the same groups. The current trial is the first RCT trial which have separate study arms for the patients with early loss of alignment. The authors are aware of the inclusion criteria of the Arora’s work.*

2.      Methods. "Gustilo 1 gradus" should be "Gustilo Grade 1" and should be referenced.

***Response:*** *Thank you for the comment. The sentence has now been amended as: “Open fracture more than Gustilo Grade 1”*

3.      Methods, outcomes. Was the outcome "the number of concomitant surgeries" measuring true "concomitant" (meaning "at the same time") surgeries, or subsequent surgeries?

***Response:*** *Thank you for the comment. The authors have meant subsequent surgeries, and the sentence has now been amended as: “the number of subsequent surgeries”*

4.      Methods. Stats. The statement "We estimated group absolute risk difference using logistic regression" seems wrong. Logistic regression provides odds ratios, not absolute risk differences.

***Response:*** *Logistic regression provides odds ratios, but the logistic model can be used to calculate marginal probability. This provided a very convenient way to assess adjusted absolute risk difference between the two exposure variables.
Please see eg.:* [*https://www.r-bloggers.com/2021/06/estimating-a-risk-difference-and-confidence-intervals-using-logistic-regression/*](https://www.r-bloggers.com/2021/06/estimating-a-risk-difference-and-confidence-intervals-using-logistic-regression/) *and* [*https://www.sciencedirect.com/science/article/abs/pii/S0895435610000454*](https://www.sciencedirect.com/science/article/abs/pii/S0895435610000454)

5.      Outcome scores. These needs better explanation. I have never heard of the "15-D" and there is no reference provided for this or any other outcomes, and no information on the range and whether higher scores are better or worse.

***Response:*** *Thank you for this important notice. All outcome variables are explained in the supplementary material (S11 Trial Metadata), as the manuscript text has a strict word limit. There have also been added references for those who want to read more about the outcomes of this trial. In the manuscript, there has been added a mention of where to find the information: “Detailed information of the outcome variables can be found in the supplementary material (S11 Trial Metadata).”*

6.      Discussion. I find sentences like this to be unnecessary and misleading: "Still, according to 95% confidence intervals, we cannot definitively rule out the benefit of operative treatment". It is also true that you cannot rule out the benefit of non-operative treatment (for the late malalignment group referenced). I would remove this sentence. The readers can judge the 95% CI for themselves.

***Response:*** *Thank you for this comment. The sentence has now been removed, as suggested.*

7.      Discussion. Similarly, I find statements like this to be misleading: "the nonoperatively treated groups had wider variances; meaning that they had more patients with poor outcomes". If they had wider variances, did they not also have more patients with very good outcomes? I think this line of argument is measuring things that were not intended to be measured, and only measuring one side of the argument is biased. I would remove this section.

***Response:*** *Thank you for this comment.* *The statement* “*the nonoperatively treated groups had wider variances; meaning that they had more patients with poor outcomes” is a finding from meta-analysis by Linnanmäki et al, and it is not an interpretation of the authors of this manuscript.* *In addition, in the present study, the distributions of PRWE are strongly positively skewed, and the difference between groups comes mainly in the number of worse results, which is seen as an increase in the variance. The authors think this is an important finding in a clinical sense, as these are the patients who make the difference between treatment results of operative and non-operative treatment, even though the estimated mean effect is below the MCID. The authors would like to keep this section as it is now, unless the Editor thinks that this section should be removed.*

8.      Discussion. The authors again argue that the "key gap" in the literature is patients with loss of alignment after initial reduction. This means they have not read the inclusion criteria in the supplement of the Arora et al study that they reference. Same for their statement further down "To our knowledge, this is the first trial…" and in line 414 "Out study is the first…". This is always a dangerous thing to claim.

***Response:*** *Thank you for the comment. The authors are aware of the inclusion criteria of Arora et al. and the criteria were checked when planning the current trial. Even though Arora et al. in their randomized study included patients who lost fracture alignment in the early follow-up, the patients were analyzed in the same group as other operatively or non-operatively treated patients, which differs from the current trial. There are also other previous trials that have included patients with early loss of alignment, but they have not randomized patients to receive surgery or to continue non-operative treatment, e.g. the study from Sirniö et al. (Sirniö K, Leppilahti J, Ohtonen P, Flinkkilä T. Early palmar plate fixation of distal radius fractures may benefit patients aged 50 years or older: a randomized trial comparing 2 different treatment protocols. Acta orthopaedica. 2019. p. 123‐128. doi:10.1080/17453674.2018.1561614). However, after those trials, we still do not have enough knowledge to answer the question whether we should offer patients operative treatment in case of early loss of alignment. To make the difference between current trial and previous trials more clear, this statement has now been amended as follows: “To our knowledge, this is the first trial to have separate randomization and separate study arms to evaluate treatment effect between operative and non-operative treatment in patients with early loss of alignment of DRF.”*

9.      Discussion. There is too much repetition of results in the discussion. It is not necessary to repeat the kg strength measures, the 95% CIs, table references, etc in the discussion as the results have already been provided in the Results section. Talk ABOUT the results, don't repeat them unless absolutely necessary (e.g., comparing the difference found with the MCID)

***Response:*** *Thank you for the comment. The authors have aimed to reduce unnecessary repetition when revising the discussion of the manuscript.*

10.     It is not allowed to present NEW results in the discussion (like the difference in effect between age groups). This looks like it came from Supplement 6, which also includes the per protocol analysis. These things should be provided in the Results, even if it is just a reference to the supplement.

***Response:*** *Thank you for the important notice. There has now been added a mention in the Results section, that the subgroup analysis can be found in supplementary material S6: “Subgroup analysis by age can be found in the supplementary material (S6 Additional analyses).”*

11.     Discussion, line 390. It is not possible for fractures to "dislocate". Perhaps the authors meant "displace" or "lose alignment"?

***Response:*** *Thank you for this comment. The authors have meant loss of alignment, and the sentence has now been amended as “have been reported to be risk factors that predispose loss of alignment of the DRF”*

12.     Discussion, line 411. How did the change in local treatment "towards more non-operative treatment" lead to "more patients … undergoing surgical treatment". This seems contradictory.

***Response:*** *Thank you for this notice. The sentence has now been amended as “Furthermore, a change in local treatment practices towards more nonoperative treatment led to more patients declining to join the study, which could have led to undergoing surgical treatment.”*

Reviewer #3: Statistical review
This paper reports a trial that compared volar locking plating vs non-operative treatment at two points of a patient's treatment for distal radius fracture. I thought that the statistical methods and results were generally reported well and only had some minor comments.

***Response:*** *The authors thank you for the positive feedback.*

1.      Abstract and results: for the primary outcome, I would recommend a p-value is given in addition to confidence interval. This would seem appropriate given that the trial was powered to test a null hypothesis of no difference (and appears consistent with the SAP).

***Response:*** *Thank you for the comment. P values have now been provided for the primary outcome (PRWE at 12 months), as suggested.*

2.      Abstract: although the point estimate was lower than the MCID, the uncertainty intervals do seem consistent with the MCID being plausible. I would recommend adding 'estimated' before 'mean difference' if the authors wish to retain this point.

***Response:*** *Thank you for this comment. We have now amended the term as “estimated mean difference”.*

3.      Outcome variables, page 9: I note in the SAP and the CT.gov page, the plan was to include 2-year data in the analysis. Was this abandoned due to the trial finishing early, or will the 2 year results be reported elsewhere?

***Response:*** *Thank you for the important notice. The results reported in this manuscript were obtained after finishing the 12-month follow-up of the trial and after analyzing the data collected until then. The 2-year results of the trial will be reported later.*

4.      Sample size, page 9: I assume that from the '95% CI' mentioned, that the type I error rate was 5%, two-sided - I would recommend that this is added. It may also be useful to clarify that no adjustment for multiple testing from the two main comparisons was made.

***Response:*** *Thank you for these great comments. The mentions of type I error rate of 5%, two-sided, and the fact that no adjustment for multiple testing from the two main comparisons was made have now been added in the Methods, Statistical analysis section.*

5.      Statistical analysis, page 9: for the linear mixed effects model, it would be useful to briefly give what fixed and random effects were used. For instance, was baseline PRWE adjusted for?

***Response:*** *Unfortunately, we did not measure baseline PRWE. In the LME, patient was a random factor, age and intra/extra articularity of the fracture were fixed.*

6.      Statistical analysis, page 9: I would briefly mention how missing outcome data was handled (presumably it was included in the linear mixed effects model, which would be valid under a MAR assumption). The proportion of participants who were lost to follow-up was much lower than assumed, so this issue isn't too important!

***Response:*** *We did not impute any data. No case was excluded unless it missed all outcome data.*

7.      Secondary outcomes: although all the results are presented in the table, it may be useful to have a brief narrative of which secondary results were significant and which weren't.

***Response:*** *Thank you for the comment. The methods section, outcomes has now been amended to have a mention of secondary outcomes exceeding MCID:
-Primarily malaligned DRFs: “At 12 months, the mean effect for grip strength was 5.3 kgs (95% CI [2.9, 7.7]) and for 15-D 0.017 (95% CI [-0.001, 0.04]), both exceeding the level of MCID. Detailed results for other secondary outcomes are presented in Table 2.”*

*-Early malaligned DRFs: At 12 months, the mean effect for grip strength was 5.3 kgs (95% CI [3.2, 7.4]) reaching the level of MCID.“*

-----------------------------------------------------------

General editorial requests:
(Note: not all will apply to your paper, but please check each item carefully)

*       We ask every co-author listed on the manuscript to fill in a contributing author statement, making sure to declare all competing interests. If any of the co-authors have not filled in the statement, we will remind them to do so when the paper is revised. If all statements are not completed in a timely fashion this could hold up the re-review process. If new competing interests are declared later in the revision process, this may also hold up the submission. Should there be a problem getting one of your co-authors to fill in a statement we will be in contact. Please do not add or remove authors without first discussing this with the handling editor. You can see our competing interests policy here: <https://eur04.safelinks.protection.outlook.com/?url=http%3A%2F%2Fjournals.plos.org%2Fplosmedicine%2Fs%2Fcompeting-interests&data=05%7C02%7Cteemu.hevonkorpi%40tuni.fi%7C0649261777bc42f353c208ddc3b80c7d%7Cfa6944afcc7c4cd89154c01132798910%7C0%7C0%7C638881917479040267%7CUnknown%7CTWFpbGZsb3d8eyJFbXB0eU1hcGkiOnRydWUsIlYiOiIwLjAuMDAwMCIsIlAiOiJXaW4zMiIsIkFOIjoiTWFpbCIsIldUIjoyfQ%3D%3D%7C0%7C%7C%7C&sdata=f3u8PFjAygWwxzkPMolhBZGRyE2HfPstT4SEzR1bDBM%3D&reserved=0>.

***Response:*** *Thank you for the information.*

*       Please upload any figures associated with your paper as individual TIF or EPS files with 300dpi resolution at resubmission; please read our figure guidelines for more information on our requirements:
<https://eur04.safelinks.protection.outlook.com/?url=http%3A%2F%2Fjournals.plos.org%2Fplosmedicine%2Fs%2Ffigures&data=05%7C02%7Cteemu.hevonkorpi%40tuni.fi%7C0649261777bc42f353c208ddc3b80c7d%7Cfa6944afcc7c4cd89154c01132798910%7C0%7C0%7C638881917479064636%7CUnknown%7CTWFpbGZsb3d8eyJFbXB0eU1hcGkiOnRydWUsIlYiOiIwLjAuMDAwMCIsIlAiOiJXaW4zMiIsIkFOIjoiTWFpbCIsIldUIjoyfQ%3D%3D%7C0%7C%7C%7C&sdata=L4ny6f%2BecTtOsDyu3mOCIjfOwjbcwt6eiKV%2FB87pbRs%3D&reserved=0>. While revising your submission, please upload your figure files to the PACE digital diagnostic tool, 
<https://eur04.safelinks.protection.outlook.com/?url=https%3A%2F%2Fpacev2.apexcovantage.com%2F&data=05%7C02%7Cteemu.hevonkorpi%40tuni.fi%7C0649261777bc42f353c208ddc3b80c7d%7Cfa6944afcc7c4cd89154c01132798910%7C0%7C0%7C638881917479080154%7CUnknown%7CTWFpbGZsb3d8eyJFbXB0eU1hcGkiOnRydWUsIlYiOiIwLjAuMDAwMCIsIlAiOiJXaW4zMiIsIkFOIjoiTWFpbCIsIldUIjoyfQ%3D%3D%7C0%7C%7C%7C&sdata=R9tmRUJXxj%2FlJdL66Smykw8MXBKspUHoMoQYFapDdYw%3D&reserved=0>. PACE helps ensure that figures meet PLOS requirements. To use PACE, you must first register as a user. Then, login and navigate to the UPLOAD tab, where you will find detailed instructions on how to use the tool. If you encounter any issues or have any questions when using PACE, please email us at [PLOSMedicine@plos.org](mailto:PLOSMedicine@plos.org).

***Response:*** *Thank you for the information. Fig1 and Fig2 have now been uploaded as TIF files. We encountered technical problems when we tried to use the PACE tool. The website does not work and has a text “502 - Web server received an invalid response while acting as a gateway or proxy server.” We tried to use it with multiple different devices, but still did not manage to get it work. However, in the author guidelines there was a suggestion to use the NAAS tool, and Fig1 and Fig2 were edited with that tool.*

*       Please ensure that the paper adheres to the PLOS Data Availability Policy (see <https://eur04.safelinks.protection.outlook.com/?url=http%3A%2F%2Fjournals.plos.org%2Fplosmedicine%2Fs%2Fdata-availability&data=05%7C02%7Cteemu.hevonkorpi%40tuni.fi%7C0649261777bc42f353c208ddc3b80c7d%7Cfa6944afcc7c4cd89154c01132798910%7C0%7C0%7C638881917479094221%7CUnknown%7CTWFpbGZsb3d8eyJFbXB0eU1hcGkiOnRydWUsIlYiOiIwLjAuMDAwMCIsIlAiOiJXaW4zMiIsIkFOIjoiTWFpbCIsIldUIjoyfQ%3D%3D%7C0%7C%7C%7C&sdata=xHLR8ZdR2wq981YLr6rdde3kutrvGsSMuR3cp4oLTsU%3D&reserved=0)>, which requires that all data underlying the study's findings be provided in a repository or as Supporting Information. For data residing with a third party, authors are required to provide instructions with contact information (web or email address) for obtaining the data. Please note that a study author cannot be the contact person for the data. PLOS journals do not allow statements supported by "data not shown" or "unpublished results." For such statements, authors must provide supporting data or cite public sources that include it.

***Response:*** *Thank you for the information. The data sharing statement with contact information is included in the manuscript, as instructed.*

*       We expect all researchers with submissions to PLOS in which author-generated code underpins the findings in the manuscript to make all author-generated code available without restrictions upon publication of the work. In cases where code is central to the manuscript, we may require the code to be made available as a condition of publication. Authors are responsible for ensuring that the code is reusable and well documented. Please make any custom code available, either as part of your data deposition or as a supplementary file. Please add a sentence to your data availability statement regarding any code used in the study, e.g. "The code used in the analysis is available from Github [URL] and archived in Zenodo [DOI link]" Please review our guidelines at <https://eur04.safelinks.protection.outlook.com/?url=https%3A%2F%2Fjournals.plos.org%2Fplosmedicine%2Fs%2Fmaterials-software-and-code-sharing&data=05%7C02%7Cteemu.hevonkorpi%40tuni.fi%7C0649261777bc42f353c208ddc3b80c7d%7Cfa6944afcc7c4cd89154c01132798910%7C0%7C0%7C638881917479107455%7CUnknown%7CTWFpbGZsb3d8eyJFbXB0eU1hcGkiOnRydWUsIlYiOiIwLjAuMDAwMCIsIlAiOiJXaW4zMiIsIkFOIjoiTWFpbCIsIldUIjoyfQ%3D%3D%7C0%7C%7C%7C&sdata=e%2FcCLRcENXlALvTTEEW3G83CYM6eZWAQg47pb1i9hgo%3D&reserved=0> and ensure that your code is shared in a way that follows best practice and facilitates reproducibility and reuse. Because Github depositions can be readily changed or deleted, we encourage you to make a permanent DOI'd copy (e.g. in Zenodo) and provide the URL.

***Response:*** *No author generated code was used.*

FORMATTING - GENERAL
*       Abstract: Please structure your abstract using the PLOS Medicine headings (Background, Methods and Findings, Conclusions). Please combine the Methods and Findings sections into one section.

***Response:*** *The abstract has been structured according to the formatting guidelines.*

*       At this stage, we ask that you include a short, non-technical Author Summary of your research to make findings accessible to a wide audience that includes both scientists and non-scientists. The Author Summary should immediately follow the Abstract in your revised manuscript. This text is subject to editorial change and should be distinct from the scientific abstract. Ideally each sub-heading should contain 2-3 single sentence, concise bullet points containing the most salient points from your study. In the final bullet point of 'What Do These Findings Mean?', please include the main limitations of the study in non-technical language. Please see our author guidelines for more information: <https://eur04.safelinks.protection.outlook.com/?url=https%3A%2F%2Fjournals.plos.org%2Fplosmedicine%2Fs%2Frevising-your-manuscript%23loc-author-summary&data=05%7C02%7Cteemu.hevonkorpi%40tuni.fi%7C0649261777bc42f353c208ddc3b80c7d%7Cfa6944afcc7c4cd89154c01132798910%7C0%7C0%7C638881917479120748%7CUnknown%7CTWFpbGZsb3d8eyJFbXB0eU1hcGkiOnRydWUsIlYiOiIwLjAuMDAwMCIsIlAiOiJXaW4zMiIsIkFOIjoiTWFpbCIsIldUIjoyfQ%3D%3D%7C0%7C%7C%7C&sdata=uGSy2TZbN%2B%2BpzMrnVkaTSKOx6Q6q%2B8Jpy9DNyrwW4LI%3D&reserved=0>.

***Response:*** *Thank you for the comment. The Author Summary has now been added to sum up the most important aspects of the trial, including the main limitations.*

*       Please express the main results with 95% CIs as well as p values. When reporting p values please report as p<0.001 and where higher as the exact p value p=0.002, for example. Throughout, suggest reporting statistical information as follows to improve clarity for the reader "22% (95% CI [13%,28%]; p</=)". Please be sure to define all numerical values at first use.

***Response:*** *Thank you for the comments. P values have now been provided for the primary outcome, as suggested.*

*       Please include page numbers and line numbers in the manuscript file. Use continuous line numbers (do not restart the numbering on each page).

***Response:*** *Page numbers and line numbers are included according to the guidelines.*

*       Please cite the reference numbers in square brackets. Citations should precede punctuation.

***Response:*** *It has been double-checked that the reference numbers are cited in square brackets and precede punctuation.*

FIGURES AND TABLES
*       Please provide titles and legends for all figures and tables (including those in Supporting Information files).

***Response:*** *Titles and legends have been provided for all figures and tables.*

*       Please define all abbreviations used in each figure/table (including those in Supporting Information files).

***Response:*** *Abbreviations have been defined as suggested.*

*       Please consider avoiding the use of red and green in order to make your figure more accessible to those with color blindness.

***Response:*** *Thank you for the comment. The colors have been amended to make the Figures accessible also to persons with color blindness.*

SUPPLEMENTARY MATERIAL
*       Please note that supplementary material will be posted as supplied by the authors. Therefore, please amend it according to the relevant comments outlined here.

***Response:*** *Thank you for the information.*

*       Please cite your Supporting Information as outlined here: <https://eur04.safelinks.protection.outlook.com/?url=https%3A%2F%2Fjournals.plos.org%2Fplosmedicine%2Fs%2Fsupporting-information&data=05%7C02%7Cteemu.hevonkorpi%40tuni.fi%7C0649261777bc42f353c208ddc3b80c7d%7Cfa6944afcc7c4cd89154c01132798910%7C0%7C0%7C638881917479134215%7CUnknown%7CTWFpbGZsb3d8eyJFbXB0eU1hcGkiOnRydWUsIlYiOiIwLjAuMDAwMCIsIlAiOiJXaW4zMiIsIkFOIjoiTWFpbCIsIldUIjoyfQ%3D%3D%7C0%7C%7C%7C&sdata=dHmDMyTKf%2BQsuasSjKTfT4KTJyGVSwPWzWWSbCHdFrU%3D&reserved=0>

***Response:*** *The supporting informations has been cited, as outlined in the guidelines.*

REFERENCES
*       PLOS uses the numbered citation (citation-sequence) method and first six authors, et al.

***Response:*** *The numbered citation has been used according to the guidelines of PLOS Medicine.*

*       Please ensure that journal name abbreviations match those found in the National Center for Biotechnology Information (NCBI) databases (<https://eur04.safelinks.protection.outlook.com/?url=http%3A%2F%2Fwww.ncbi.nlm.nih.gov%2Fnlmcatalog%2Fjournals&data=05%7C02%7Cteemu.hevonkorpi%40tuni.fi%7C0649261777bc42f353c208ddc3b80c7d%7Cfa6944afcc7c4cd89154c01132798910%7C0%7C0%7C638881917479147438%7CUnknown%7CTWFpbGZsb3d8eyJFbXB0eU1hcGkiOnRydWUsIlYiOiIwLjAuMDAwMCIsIlAiOiJXaW4zMiIsIkFOIjoiTWFpbCIsIldUIjoyfQ%3D%3D%7C0%7C%7C%7C&sdata=j1yUP5EZzHii%2FOrb8a7hnJpfn0ehuVvUv1iLoN0rOcc%3D&reserved=0>), and are appropriately formatted and capitalised.

***Response:*** *Thank you, this has been ensured.*

*       Where website addresses are cited, please include the complete URL and specify the date of access (e.g. [accessed: 12/06/2023]).

***Response:*** *Thank you, no website addresses were cited.*

*       Please also see
<https://eur04.safelinks.protection.outlook.com/?url=https%3A%2F%2Fjournals.plos.org%2Fplosmedicine%2Fs%2Fsubmission-guidelines%23loc-references&data=05%7C02%7Cteemu.hevonkorpi%40tuni.fi%7C0649261777bc42f353c208ddc3b80c7d%7Cfa6944afcc7c4cd89154c01132798910%7C0%7C0%7C638881917479160242%7CUnknown%7CTWFpbGZsb3d8eyJFbXB0eU1hcGkiOnRydWUsIlYiOiIwLjAuMDAwMCIsIlAiOiJXaW4zMiIsIkFOIjoiTWFpbCIsIldUIjoyfQ%3D%3D%7C0%7C%7C%7C&sdata=DNVSP%2BGU%2FAr8UY0cDMLEkIwhCI0N%2FkjjLHEN3tkJh%2FE%3D&reserved=0> for further details on reference formatting.

***Response:*** *Thank you, the details for reference formatting were checked thoroughly.*

STUDY TYPE-SPECIFIC REQUESTS

*       PLOS Medicine requires that all trials be prospectively registered in one of registries recognized by WHO. Please ensure that study registration details are included in the Methods section.

***Response:*** *The registration details of the current trial are included in the Methods section.*

*       Please structure the Methods section using the following sub-headings: Study design and participants, Randomization and masking, Procedures, Outcomes, Statistical analysis.

***Response:*** *The Methods section has now been amended to include the sub-headings as requested.*

*       Please clarify and explain all discrepancies between the paper and protocol. We have pointed out some instances below. If outcomes were not prespecified in the protocol, please define them in the Methods (Outcomes section) as post hoc and explain why they were added. Post-hoc comparisons should be presented as hypothesis generating rather than conclusive.
- The following outcome measure, "The primary outcome in this study is the PRWE score measured after one and two years.", appears to differ between the submitted manuscript and the protocol [and/or trial registry]. Please clarify.

***Response:*** *Thank you for the important comment. The PRWE was chosen as the primary outcome when planning the trial. To make sure that the follow-up is long enough, we decided to have a 24-month follow-up. As there can be only one primary outcome, it was decided to be 12 months of PRWE. The follow-up was continued to 24-months as planned, and the 24-months results will be reported later when we have collected and analyzed all the data. The authors admit that in the Research plan of the trial, this has been written unclearly.*

- In the protocol, you have listed "the number of wrist movements measured with Axivity accelerometer" as a secondary outcomes. However, this does not seem to be reported in the submitted manuscript. Please clarify.

***Response:*** *The Axivity accelerometer data is collected, but we have not managed to analyze all the data thus far. The whole concept of using accelerometer in the follow-up of the patients was seen that complex, that the authors decided, that the results of the accelerometer follow-up would be better to report separately from the main results, the patient-rated outcome measures.*

- In the manuscript, you listed grip strength and adverse events as secondary outcomes, which appears to differ from the protocol. Please clarify.

***Response:*** *Thank you for this important comment. Grip strength and adverse events were both secondary outcomes of the trial. In the protocol the secondary outcomes are listed as follows: “The secondary outcomes measured are disability [Quick-DASH (disabilities of the arm, shoulder and arm)], pain in visual analogue scale (VAS), PCS, quality of life (15-D), physical activity and the number of wrist movements measured with Axivity accelerometer,* ***complications*** *and the number of surgical interventions in the conservatively managed group.” As you pointed out, grip strength appears to be missing from that listing. However, it is stated in the procotol in sections 1.OBJECTIVES: “To compare pain, disability, quality of life,* ***grip strength*** *and the number of complications”, in section 2. PRIMARY HYPOTHESIS “Initial or delayed operative treatment of malaligned distal radius fracture does not result in superior results compared with non-operative treatment with regard to pain, disability, quality of life,* ***grip strength****, and number of complications”, and in section 4.4 FOLLOW-UP “****Grip strength*** *will also be measured.” The authors apologize that grip strength was mistakenly missing from the list of the protocol section 3. STUDY DESIGN.*

*       Please ensure that all prespecified outcomes (primary, secondary, and exploratory) are listed in the Methods/Outcomes section and indicate whether there are outcomes that are not presented in the current report.

***Response:*** *Thank you for this valuable comment. All prespecified outcomes are now listed in the Methods/Outcomes section and we added the text about outcomes which are not reported in the current paper and will be reported later when the data of them has been analyzed “The Axivity accelerometer data, and the data of Edinburgh Wrist Calculator are not reported in this publication and will be reported afterwards .”*

*       Please specify the dates (Month Day, Year) during which study enrollment and follow up occurred.

***Response:*** *The dates during which the trial enrollment and follow-up occurred have been specified as follows: “Between March 15, 2018 and June 6, 2023, 291 patients aged 65 years or older who had sustained a DRF were included in the trial. The 12-month follow-up was completed on June 11, 2024.”*

*       Please include absolute numbers wherever you report percentages; eg, n/N (%)

***Response:*** *Thank you for the instruction. Absolute numbers have now been added in the “n/N (%)” format in Table 1 and Table 3.*

*       Please present the safety data for the study including numbers of specific events and whether or not adverse events are thought to be related to treatment. AEs should be reported in the abstract, per CONSORT and CONSORT-Harms.

***Response:*** *Thank you for the comment. The adverse events have now been classified as treatment-related adverse events or adverse events unrelated to received treatment in Table 3 of the manuscript. There has also been added a mention of adverse events in the abstract “At 12-month follow-up, we found 25 treatment-related adverse events (10/66, 15% in primarily malaligned DRFs non-operative; 5/58, 8.6% in primarily malaligned DRFs operative; 2/44, 4.5% in early malaligned DRFs non-operative; 3/42, 7.1% in early malaligned DRFs operative; 5/63, 7.9% in well-aligned DRFs) and 11 other adverse events.”*

*       Please complete the CONSORT checklist (<https://eur04.safelinks.protection.outlook.com/?url=https%3A%2F%2Fwww.equator-network.org%2Freporting-guidelines%2Fconsort%2F&data=05%7C02%7Cteemu.hevonkorpi%40tuni.fi%7C0649261777bc42f353c208ddc3b80c7d%7Cfa6944afcc7c4cd89154c01132798910%7C0%7C0%7C638881917479177042%7CUnknown%7CTWFpbGZsb3d8eyJFbXB0eU1hcGkiOnRydWUsIlYiOiIwLjAuMDAwMCIsIlAiOiJXaW4zMiIsIkFOIjoiTWFpbCIsIldUIjoyfQ%3D%3D%7C0%7C%7C%7C&sdata=FFSq0pv0F1gFS2dtG6Eyy1wXFoB6RArqpmtHScgUtH4%3D&reserved=0>) and ensure that all components of CONSORT are present in the manuscript, including how randomization was performed, allocation concealment, blinding of intervention, definition of lost to follow-up, power statement. When completing the checklist, please use section and paragraph numbers, rather than page numbers.

***Response:*** *Thank you for the comment. The CONSORT checklist (S1 DRIFT TRIAL CONSORT 2025 Checklist) has been updated to have correct information after manuscript revision.*

*       Please report your abstract according to CONSORT for abstracts, following the PLOS Medicine abstract structure (Background, Methods and Findings, Conclusions) <https://eur04.safelinks.protection.outlook.com/?url=https%3A%2F%2Fwww.equator-network.org%2Freporting-guidelines%2Fconsort-abstracts%2F&data=05%7C02%7Cteemu.hevonkorpi%40tuni.fi%7C0649261777bc42f353c208ddc3b80c7d%7Cfa6944afcc7c4cd89154c01132798910%7C0%7C0%7C638881917479194065%7CUnknown%7CTWFpbGZsb3d8eyJFbXB0eU1hcGkiOnRydWUsIlYiOiIwLjAuMDAwMCIsIlAiOiJXaW4zMiIsIkFOIjoiTWFpbCIsIldUIjoyfQ%3D%3D%7C0%7C%7C%7C&sdata=V%2BNeXfm5IfP%2FXAxB5KlSs7CvhUE0uYTH2I%2BqJt%2F3TtM%3D&reserved=0>

***Response:*** *The abstract has now been amended to follow the instructions of the CONSORT for abstracts. The following amendments were done:
Methods and findings:
-Text added: “Participants and orthopaedic investigators were not blinded because the interventions were so different. The statisticians and investigators responsible for the analysis remained blinded to the treatment groups during data analysis and the drawing of conclusions.”*

*-Text amended: “Between March 15, 2018 and June 6, 2023, 291 patients aged 65 years or older (mean age 73 years (SD 5.8), 258 women, 33 men) who had sustained a DRF were included in the trial.”*

*-Text added: “The 12-month follow-up was completed on June 11, 2024.”*

*-Text added: “At 12-month follow-up, we found 25 treatment-related adverse events (10/66, 15% in primarily malaligned DRFs non-operative; 5/58, 8.6% in primarily malaligned DRFs operative; 2/44, 4.5% in early malaligned DRFs non-operative; 3/42, 7.1% in early malaligned DRFs operative; 5/63, 7.9% in well-aligned DRFs) and 11 other adverse events (AE).”*

*Conclusions:
-Word “Estimated” added before the term mean difference.*

*       If your trial had to undergo important modifications in response to extenuating circumstances, please complete the CONSERVE-CONSORT checklist and provide in your Supporting Information; (<https://eur04.safelinks.protection.outlook.com/?url=https%3A%2F%2Fwww.equator-network.org%2Freporting-guidelines%2Fguidelines-for-reporting-trial-protocols-and-completed-trials-modified-due-to-the-covid-19-pandemic-and-other-extenuating-circumstances-the-conserve-2021-statement%2F&data=05%7C02%7Cteemu.hevonkorpi%40tuni.fi%7C0649261777bc42f353c208ddc3b80c7d%7Cfa6944afcc7c4cd89154c01132798910%7C0%7C0%7C638881917479208904%7CUnknown%7CTWFpbGZsb3d8eyJFbXB0eU1hcGkiOnRydWUsIlYiOiIwLjAuMDAwMCIsIlAiOiJXaW4zMiIsIkFOIjoiTWFpbCIsIldUIjoyfQ%3D%3D%7C0%7C%7C%7C&sdata=eihsGGwNfzQJKQoi%2B2A4cI%2BMo31wMUD8F13BPyJhB2E%3D&reserved=0>). When completing the checklist, please use section and paragraph numbers, rather than page numbers.

***Response:*** *Thank you for the information. We have now added the CONSERVE-CONSORT checklist as supporting information, S12 DRIFT-trial CONSERVE-Checklist*

*       In keeping with our commitment to Open Science, please include the study protocol document and analysis plan (including any amendments) as Supporting Information to be published with the manuscript if accepted.

***Response:*** *The trial protocol document and statistical analysis plan are included as Supporting Information, thank you.*

*       Please note that PLOS Medicine requires prospective, public registration of a data sharing plan (as part of mandatory clinical trials registration) for all clinical trials that began enrollment on or after January 1, 2019, in accordance with ICMJE requirements.

***Response:*** *Thank you for the information. The recruitment for the DRIFT trial was started before January 1, 2019.*

-----------------------------------------------------------

Technical requests for the revision

Journal Requirements:

1) Please provide separate figure files in .tif or .eps format. For more information about figure files please see our guidelines:

<https://eur04.safelinks.protection.outlook.com/?url=https%3A%2F%2Fjournals.plos.org%2Fplosmedicine%2Fs%2Ffigures&data=05%7C02%7Cteemu.hevonkorpi%40tuni.fi%7Cb380f2e2b65d46f93fc608ddc5131278%7Cfa6944afcc7c4cd89154c01132798910%7C0%7C0%7C638883407882472194%7CUnknown%7CTWFpbGZsb3d8eyJFbXB0eU1hcGkiOnRydWUsIlYiOiIwLjAuMDAwMCIsIlAiOiJXaW4zMiIsIkFOIjoiTWFpbCIsIldUIjoyfQ%3D%3D%7C0%7C%7C%7C&sdata=kESQRbh9BXib4dy3kvTJwV8wa9O3JLw2YWMF0DMf7AU%3D&reserved=0>

***Response:*** *Thank you for the information.The figures Fig1 and Fig2 are now provided as separate files in .tif format.*

2) We have noticed that you have uploaded Supporting Information files, but you have not included a list of legends. Please add a full list of legends for your Supporting Information files after the references list.

***Response:*** *Thank you for the comment. Supporting information* *files legends are provided after the Reference list. If there should be more detailed information included in the list, please let us know.*

3) File S2: Please confirm (a) that you are the photographer; or (b) provide written permission from the photographer to publish the photo(s) under our CC BY 4.0 license.

***Response:*** *Thank you for this important notice. Written permission to publish the material under CC BY 4.0 license has now been provided.*
